# Protective Effects of Intestinal Gallic Acid in Neonatal Dairy Calves Against Extended-Spectrum β-lactamase Producing Enteroaggregative *Escherichia coli* Infection: Modulating Intestinal Homeostasis and Colitis

**DOI:** 10.3389/fnut.2022.864080

**Published:** 2022-03-24

**Authors:** Zhiyuan He, Yulin Ma, Xu Chen, Shuai Liu, Jianxin Xiao, Yajing Wang, Wei Wang, Hongjian Yang, Shengli Li, Zhijun Cao

**Affiliations:** State Key Laboratory of Animal Nutrition, College of Animal Science and Technology, China Agricultural University, Beijing, China

**Keywords:** neonatal dairy calf, extended-spectrum β-lactamase producing *E. coli*, enteroaggregative *E. coli* (EAEC), Gallic acid, colitis, hindgut microbiome, fecal metabolome, fecal microbiota transplantation

## Abstract

Calf diarrhea induced by enteroaggregative *E. coli* (EAEC) spreads fast among young ruminants, causing continuous hazard to dairy industry. Antimicrobial drug abuse aggravates the incidence rate of multi-drug resistant (MDR) extended-spectrum β-lactamase-producing *E. coli* (ESBL-EC). However, knowledge of detection and significance of disease-related biomarkers in neonatal female calves are still limited. Gallic acid (GA), a natural secondary metabolite mostly derived from plants, has attracted increasing attention for its excellent anti-inflammatory and anti-oxidative properties. However, it is vague how GA engenders amelioration effects on clinical symptoms and colitis induced by ESBL-EAEC infection in neonatal animals. Here, differentiated gut microbiome and fecal metabolome discerned from neonatal calves were analyzed to ascertain biomarkers in their early lives. Commensal *Collinsella* and *Coriobacterium* acted as key microbial markers mediating colonization resistance. In addition, there exists a strongly positive relation between GA, short-chain fatty acid (SCFA) or other prebiotics, and those commensals using random forest machine learning algorithm and Spearman correlation analyses. The protective effect of GA pretreatment on bacterial growth, cell adherence, and ESBL-EAEC-lipopolysaccharide (LPS)-treated Caco-2 cells were first assessed, and results revealed direct antibacterial effects and diminished colonic cell inflammation. Then, oral GA mediated colitis attenuation and recovery of colonic short-chain fatty acid (SCFA) productions on neonatal mice peritonitis sepsis or oral infection model. To corroborate this phenomenon, fecal microbiota transplantation (FMT) method was adopted to remedy the bacterial infection. Of note, FMT from GA-treated neonatal mice achieved profound remission of clinical symptoms and colitis over the other groups as demonstrated by antibacterial capability and prominent anti-inflammatory abilities, revealing improved hindgut microbiota structure with enriched *Clostridia_UCG-014, Lachnospiraceae, Oscillospiraceae*, and *Enterococcaceae*, and upregulation of SCFA productions. Collectively, our findings provided the direct evidence of hindgut microbiota and intestinal metabolites, discriminating the health status of neonatal calves post ESBL-EAEC infection. The data provided novel insights into GA-mediated remission of colitis *via* amelioration of hindgut commensal structure and upregulation of SCFA productions. In addition, its eminent role as potential antibiotic alternative or synergist for future clinic ESBL-EAEC control in livestock.

## Introduction

Calf diarrhea incidence is still rising, which is mainly induced by pathogenic *E. coli*, Coronavirus, Rotavirus, *Cryptosporidium*, and some marked changes of the breeding environment (1). Recent progresses in the pathogenesis of extended-spectrum β-lactamase-producing enteroaggregative *E. coli* (ESBL-EAEC) have highlighted that specific bacterial toxins, adhesins, and siderophores coincide with host immune system disorder (2). In particular, the goal is to block bacterial adhesion to the surface of intestinal epithelial cells. It is widely accepted that ESBL-EAEC infection brings about vast changes like massive shedding, swelling, and cell apoptosis of colonic epithelium. Notably, colonic inflammation is profoundly involved in tissue injury (3). Thus, effective attenuation of colonic inflammatory responses greatly impacts prognosis post ESBL-EAEC infection. However, clinical treatment of diarrhea induced by ESBL-EAEC infection still requires chronic application of antimicrobials, which actually destroy the relative abundance and diversity of gut microbiomes and produce long-lasting adverse effects including the rapid spread of multi-drug resistance (MDR) in livestock industry (4, 5). Based on what is mentioned above, rational alternative methods targeting ESBL-EAEC and colonic inflammatory process *via* natural derived metabolites has gradually won a considerable amount of attention.

Gallic acid (GA), a naturally occurring polyphenol compound found in fruits, vegetables, and herbal medicines (6), is generally recognized as flavoring agents and preservatives with conspicuous antimicrobial, anti-inflammatory, antioxidant, and anticancer properties in the food industry (7–10). GA and its derivatives not only have extensively used in food supplementation or additives, but also regulate gut microbiome activities and immune responses (10). GA also has potent therapeutic effects on gastric mucosal damage induced by the non-steroidal anti-inflammatory drug *via* suppression of oxidative stress and mitochondrial pathway of apoptosis (11). Importantly, it is observed that GA has a broad spectrum of therapeutic properties for virus, bacterium, and fungus *in vitro*, especially the biofilm formation restraint of *Escherichia coli* (12). Due to the above-mentioned reasons, GA has been used as important raw material to synthesize trimethoprim (13). Previous studies have confirmed that GA can alleviate dextran sulfate sodium (DSS)-induced colitis in mice through the restraint of NF-κB pathway (14). Some other studies speculated that oral administration of GA could markedly increase the production of SCFA in patients with inflammatory bowel disease (IBD) (15). However, the effect of GA on colonic inflammatory responses induced by ESBL-EAEC infection and the regulatory impacts on the community structure of hindgut microbiota have not been sufficiently elucidated.

Here, healthy and diarrheal newborn female dairy calves had been involved in our study to represent young ruminants. Hindgut microbiota colonization and their fecal metabolites were strongly influenced by ESBL-EAEC infection, including the undermined GA in diarrheal feces. Based on previous publications and existing data, we inferred the therapeutic role of GA on colonic inflammation arrest which was mainly mediated by anti-bacterial capability, anti-inflammatory action, and hindgut microbiota improvement. Beneficial anti-inflammation effect of GA intervention was first investigated in the ESBL-EAEC-liposaccharide (LPS)-induced Caco-2 cellular inflammation process. Then, the alleviated effect of oral GA was further investigated in peritonitis sepsis and oral infection of neonatal CD-1 mice induced by ESBL-EAEC. More importantly, the positive effects of specific hindgut commensals in GA-treated donors had been thoroughly detected through a comparison between neonatal mice inoculated with fecal microbiota transplantation (FMT) and control groups. Herein, we tried to shed light on the potential hazards and risks of ESBL-EAEC infection among neonatal dairy calves, and suggested GA as a potential alternative or synergist for future control of ESBL-EAEC infection cases.

## Materials and Methods

### Bacterial Strains and Bacteria Culture

The mentioned strain of this study was separated from an autochthonous clinical trial, and the antibiotic susceptibility of the strain was shown in previous publication (16). Genomic information of *E. coli* 1587 was shown in Supplementary Table 1. *E. coli* K12 cells were preserved in our lab. Except for special instructions, all isolates were incubated using MacConkey agar plates (Luqiao company, Beijing, China) or Luria-Bertani broth (LB, Qingdao Hope Bio-technology) at 37°C. Antibiotics were obtained from the China Institute of Veterinary Drug Control. All *E. coli* isolates were screened for the phenotypic identification of ESBL producers on MacConkey agar containing cefotaxime (2 mg/L) prior further confirmation using double-disc synergy testing in accordance with Clinical and Laboratory Standards Institute (CLSI) recommendations. Bacterial isolates were determined to be positive when the clear zone inhibition of ceftazidime plus clavulanic acid or cefotaxime plus clavulanic acid was at least 5 mm larger than their respective single disks (17). Then, part of culture medium was taken out to dilute in fresh LB until it reaches a starting optical density at 600 nm (OD_600_) of 0.2 at a 10 ml conical tube. Filter-sterilized GA (Gallic acid, Sigma-Aldrich, CID 370, 0.08 g/L and 0.8 g/L dissolved in distilled water) were separately appended to groups and further cultured at 37°C with shaking at 200 rpm until harvest. Specifically, cultures were mixed to acquire the optical density data at the indicated time-points post treatment (4, 8, 12, and 24 h).

### Calf Health Status Assessment and Fecal Sample Collection

The animal experiment was conducted in a conventional cow pasture in Heilongjiang Province, China, following the approved protocols of the experiment by Beijing Association for Science and Technology (ID no. SYXK, 2016-0008). The animal experiment was conducted in early spring, with temperatures of 7 ± 2°C, humidity of 60 ± 5%, and light intensity of 180 ± 20 lux for 18–20 h. The neonatal Holstein female calves were separated from dams immediately after birth and transferred to individual pens, following navel disinfection with 7% iodine. No animals received medical treatment in this study and health conditions, i.e., presence or absence of disease, injury, and dehydration, were appraised daily during our experiment. The pasteurized colostrum (60°C for 60 min) was thawed at 37°C using a water bath and calves were fed 4 L of colostrum within 2 h after birth using bottle feeding in the post-natal care service. Animal rectal temperature, fecal score, and respiratory score were recorded as in previously published methods (18). Then, they were bucket fed 4 L of milk replacer daily at 2–7 days after birth (phase I) and 5 L at days 8–14 (phase II) after birth. The milk replacer contained 83.3% of skim milk powder with 260 g/kg crude protein, 160 g/kg crude fat, 10 g/kg crude ash, and 19.2 MJ/kg metabolizable energy on a dry matter basis (Nutrifeed, IN, Netherlands). Calves with diarrhea were monitored and were classified as “diarrhea” when their fecal score was ≥3 for at least 2 days and positive of diarrheagenic *E. coli* (19). On the other hand, calves of the same age were identified as “healthy” when their fecal score was ≤2 for at least 2 days and without pathogen infections (Supplementary Table 2). In this trial, no calves were treated with antibiotics. Fecal samples (~10 g) were collected directly from the rectum by rectal palpation using sterile gloves and dedicated equipment to prevent cross-contamination. Samples were then subjected to the detection of *E. coli* 1587, Rotavirus, Coronavirus, and *Cryptosporidium* antigens using PCR method or commercial ELISA kits (IDEXX, Maine, USA). Eventually, 40 samples were enrolled with phase I (10 healthy calves-H_1, 11 diarrheal calves-D_1) and phase II (10 healthy calves-H_2, 9 diarrheal calves-D_2) over this entire study. All collected fecal samples were stored at −80°C immediately after collection.

### Gut Microbiota Profiling

Fecal samples were collected, and bacterial genomic DNA extraction was performed using a QIAamp DNA Isolation Kit (Qiagen, Hilden, Germany). Amplicons of the V3-V4 region within the 16S rRNA gene were conducted by PCR using optimized primers (338F: 5′-ACTCCTACGGGAGGCAGCAG-3′, 806R: 5′-GGACTACHVGGGTWTCTAAT-3′). PCR product library was prepared using the TruSeq Nano DNA LT Library Prep Kit (Illumina, SanDiego, USA), following sequenced on the Illumina Miseq platform (2 × 300, pair end). Sequencing data were processed using QIIME2 version 2020.02 (20). Then, taxonomy was assigned to filtered amplicon sequence variants (ASVs) using a pretrained QIIME2-compatible SILVA version 132 database, with 99% identity for the bacteria and representative sequences (21). Species diversity was determined using q2-diversity (http://www.r-project.org/).

### Untargeted Metabolomics Analyses

Fecal samples (5 mg) from healthy or diarrheal calves were homogenized with zirconium oxide beads for 3 min and mixed with 145 μl extraction solution (containing 25 μl of water and 120 μl of methanol) to extract the metabolites. The samples were then homogenized for another 3 min using a high-throughput tissue disruptor and centrifuged at 1,800 g for 20 min. Acquired supernatant was transferred to a 96-well plate and mixed with 20 μl derivative reagents at 30°C for 60 min, following procedures of Eppendorf epMotion Workstation (Eppendorf Inc., Humburg, Germany). Samples were further diluted with 330 μl of ice-cold 50% methanol, stored at −20°C for 20 min, and followed by centrifuged (4,000 g for 30 min at 4°C). The supernatants were obtained for liquid chromatography-mass spectrometry (LC-MS) analysis.

The metabolite extracts from calf fecal samples were analyzed using an ultra-high performance liquid chromatography coupled to tandem mass spectrometry (UPLC-MS/MS) system (ACQUITY UPLC-Xevo TQ-S, Waters Corp., Milford, MA, USA). Chromatographic separation was performed using a BEH C18 column (2.1 mm × 100 mm, 1.7 μm, Waters). The desolvation and source temperatures were set at 500°C and 150°C, respectively. Mobile phases containing acetonitrile/isopropanol (1:1, 0.1% formic acid) and 0.1% formic acid were used as carried liquid at a constant flow rate of 0.4 ml/min.

The raw data were processed using the MassLynx software (version 4.1, Waters, Milford, MA, USA). Each sample was analyzed by ultra-performance liquid chromatography coupled to tandem mass spectrometry (UPLC-MS/MS) in both positive and negative ionization modes to acquire metabolite profiles. Analysis order of all test samples was randomized. The quality-control (QC) samples were pooled samples in which both the metabolite composition of the samples and sample matrix were mixed, and then analyzed using the same methods to evaluate the quality and variance of the acquired data. Self-developed platform iMAP (version 1.0, Metabo-Profile, Shanghai, China) was used for further statistical analyses including PCA, PLS-DA, univariate analysis, and pathway analysis.

### Cell Culture and Treatments

Human colon adenocarcinoma cell line Caco-2 (ATCC-HTB-37, Manassas, USA) was maintained in Modified Eagle medium (MEM, Gibco, NY, USA) supplemented with 10% fetal bovine serum (FBS, Gibco) and 1 × penicillin-streptomycin-L-glutamine (Gibco, 10378-016) at 37°C in 5% CO_2_ incubator. As for the adhesion assay, cells were grown in 24-well tissue culture plates until reaching 80% confluence, and then incubated with *E. coli* strains at a multiplicity of infection (MOI) of 10 post washing in MEM. Cell-associated bacteria was quantified *via* removing non-adhering bacteria, which was performed following washing and cell lysis. Then, the number of Colony Forming Unit (CFU) was determined using serial dilution method and adherence percent was calculated *via* dividing the final CFU number per ml by the initial CFU number per ml for each replicate.

Then, LPS component of *E. coli* 1587 was extracted using a Lipopolysaccharide Isolation Kit (Sigma, MAK339, St.Louis, USA) and diluted to a storage concentration of 1 mg/ml in MEM. The effect of GA on LPS-treated cells was further detected *via* adding GA at a final concentration of 0.01 g/L to normal culture medium, and the cells were then cultured for 9 h followed by LPS (10 μg/ml of final concentration) treatment for 4 h. The same volume of MEM was used as negative controls.

### Murine Treatment and Sample Collection

Littermate pregnant specific pathogen-free (SPF) CD-1 mice (Sipeifu Biotechnology, Beijing, China) were adapted to standardized environmental conditions (temperature = 25 ± 2°C; humidity = 55 ± 10%), with a 12-h cycle of light and darkness, food, and sterile water *ad libitum*. Mice maintenance was strictly observed in accordance with the rules for the Administration of Affairs Concerning Experimental Animals approved by the State Council of People's Republic of China (14-11-1988). As for neonatal mice, the day of birth was considered day 0. Female mice were screened at day 2 and verified to have ingested breast milk (abdominal milk spot). After 1 week of acclimatization, murine infection test was done by intraperitoneally injected or oral gavage with a sublethal dose of *E. coli* 1587 persisters suspension (3.0 × 10^5^ CFU per mouse). Mice were weaned at day 21. The murine experiments were approved by the institutional ethics committees of China Agricultural University (XXMBB-2012-03-15-1).

Gallic acid was dissolved in the sterile water to a concentration of 40 mg/kg body weight. Two-day-old female mice were randomly divided into three treatments with 12 mice and one dam per group. These mice received daily oral gavage of 50 μl GA or the same volume of sterile water for 14 days.

Bacterial infection route was responsible for the virulence and invasion of clinical isolates. Hence, they were firstly challenged with *E. coli* 1587 *via* intraperitoneal injection to induce peritonitis at day 0. Mice were randomly divided into three groups (*n* = 12 per group) as follows: CON group, mice were access to regular sterile water for 2 weeks without infection; Placebo group, free access to regular sterile water for 2 weeks with *E. coli* infection; GA group, daily gavage of GA with *E. coli* infection. Body weight was recorded daily, and disease activity index (DAI) was rated to appraise colitis severity from day 0 to day 7 as previously described (22). Murine eyeball blood was collected, and serum was generated by centrifugation (2,500 g for 15 min at 4°C) post euthanasia on day 3 and day 7. The colon was quickly removed for length measurement, and a 5-mm segment of hollow mid-colon was collected for further assessment. Colonic tissues were homogenized in sterile PBS for bacterial loads using CFU calculation method.

Then, another test was conducted to detect the effect of GA in oral infection model of neonatal mice. Two-day-old female mice were casually divided into four groups with six mice and one dam per group. These mice also received daily oral gavage of 50 μl GA for 14 days. Mice were then infected with *E. coli via* oral gavage to imitate the natural infection at day 0 to induce colitis. The treatment groups are as follows: CON group, daily gavage of regular sterile water normally for 2 weeks; GA group, oral GA for 2 weeks without infection; 1,587 group, daily gavage of regular sterile water for 2 weeks with *E. coli* 1,587 infection; 1,587 + GA group, oral GA for 2 weeks with *E. coli* 1,587 infection. Body weight and DAI were monitored daily from day 0 to 7, followed by anesthesia on day 7. Colon length was removed for measurement, and colonic tissues were collected for subsequent assessment. Colonic tissues were homogenized in sterile PBS for bacterial loads using CFU calculation method. Mice serum was generated by centrifugation of blood samples (2,500 g for 15 min at 4°C) which were frozen at −80°C following assessment of serum cytokines.

For the fecal transplantations, 2-day-old female mice received daily oral gavage of 50 μl GA or the same volume of sterile water alone for 14 days. Meanwhile, mice were challenged with *E. coli* 1587 *via* oral gavage at day 0. They were randomized to three groups (*n* = 4–6 per group) as follows: CON group, free access to regular sterile water for 2 weeks without infection; Placebo group, oral gavage of regular sterile water for 2 weeks, with *E. coli* 1587 infection; and GA group, oral gavage of GA daily for 2 weeks with *E. coli* 1587 infection. Fresh feces of each group were pooled and homogenized in sterile saline to a liquid solution (final concentration of 100 mg feces/ml). The fecal supernatant was obtained by centrifugation and filtration and was then orally inoculated into recipient mice. Two-day-old female mice were administered with FMT *via* oral gavage two times a day (50 μl per mouse) during 1-week period. Mice were followed with *E. coli* 1587 infection *via* oral gavage at day 8. Then, body weight and DAI were measured daily post infection. Mice fresh feces were collected sterilely during day 14 and day 15 for future assessment of microbiota profiles. All mice were sacrificed by anesthesia at day 15, and the colon was quickly collected for length measurement and future detection of colonic contents.

### Quantitative Reverse Transcription-PCR (qRT-PCR) Assay

Total RNA from Caco-2 cell or colon tissue was extracted using the EASYspin Plus kit (Aidlab, Beijing, China) following the instructions of manufacture. Total RNA was treated with Dnase?, and 500 ng of total RNA was used for cDNA synthesis by reverse transcription using the PrimeScript RT reagent Kit with gDNA Eraser (Takara, Beijing, China). The cDNA was amplified with specific primers for the indicated genes, including IL-1β, IL-6, TNF-α, IL-10, and TGF-β genes, as previously described (23, 24). The real-time PCR was performed, and products were detected using QuantStudio 6 Flex Real-Time PCR System (Applied Biosystem, CA, USA). The PCR was performed in a 20-μl volume containing 1 μl of cDNA, 10 μl of 2 × SYBR green Premix Ex Taq (TaKaRa Bio Inc., Japan), and a 0.4 μM concentration of each gene-specific primer. Thermal cycling parameters were as follows: 95°C for 2 min, followed by 42 cycles of 95°C for 15 s, 60°C for 15 s, and 72°C for 15 s, and 1 cycle of 95°C for 30 s, 60°C for 30 s, and 95°C for 30 s. The final step was to obtain a melting curve for the PCR product to determine the specificity of the amplification. Each sample was run in triplicates on the 96-well sample plate, and the expression level of each gene was calculated relative to the expression of the glyceraldehyd-3-phosphate dehydrogenase (GAPDH) gene.

### Enzyme-Linked Immunosorbent Assay

Mice serum sample was generated by centrifugation of eye-blood sample at 2,500 g for 15 min. The inflammatory state of serum was assessed by measurement of inflammatory cytokines including IL-1β and IL-10 (ABclonal Technology, Wuhan, China) according to the instructions of manufacturer.

### Histological Assessment

Colonic tissues were fixed in 10% formalin for 48 h, dehydrated, and embedded into paraffin following standard steps. About 4 μm thick paraffin sections were subjected to hematoxylin and eosin (H&E) staining. Visual images were further analyzed using the Image J software (version 1.8.0_172, National Institutes of Health, Bethesda, MD, USA). The extent of colitis (including ulcer, ulceration, crypt structure, crypt loss, and presence or absence of edema) was assessed, and the histopathology scoring was determined in a blind manner according to the previously described protocols (25).

### Measurements of SCFA Profiles

Colonic contents or fecal samples were collected for SCFA (such as acetate, propionate, and butyrate) measurement *via* gas chromatography as previously described (26). In detail, 100 mg of samples from mice were dissolved, homogenized in ultrapure water, and suspension pH was adjusted to 2–3. The clear supernatant was collected post centrifugation at 5,400 rpm for 20 min. Zero-point-two milliliters deproteinized solution of 2-ethylbutyric acid was mixed with supernatant in an ice water bath for 30 min, followed by centrifugation at 10,000 rpm for 10 min. Acquired supernatant was transferred to a 2 ml screw-cap vial, and then subjected for SCFA analysis with Gas Chromatography System (Agilent Technologies, Wilmington, Delaware, USA).

### Western Blot Analysis

Following treatments, harvested cells or colonic tissue samples used for western blotting were prepared using a lysis buffer (50 mM Tris-HCl, 150 mM NaCl, 1 mM EDTA, 1% Triton X-100, 5 mM MgCl_2_, 10% glycerol, and 1 mM PMSF). Protein contents were obtained post centrifugation (12,000 g for 10 min at 4°C), and their respective concentrations were measured using bicinchoninic acid assay (BCA; Beyotime Biotechnology, Beijing, China) following the instructions of manufacturer. Protein samples were separated using 12% sodium dodecyl sulfate-polyacrylamide gel electrophoresis (SDS-PAGE) gel and transferred onto polyvinylidene difluoride membranes. Non-specific bindings were blocked using 5% nonfat milk at 37°C for about 1 h. Then, the membranes were incubated with the primary antibodies, followed by incubation with peroxidase-conjugated secondary antibody at 37°C for 1 h. Finally, the bands were visualized using enhanced chemiluminescence kit (Beyotime Biotechnology, Beijing, China). The antibodies used in the western blotting assays were shown as follows: phospho-IκBα (Ser36) antibody (Abcam, ab133462), IκBα antibody (Abcam, ab32518), β-actin antibody (ABclonal Technology, AC004), occludin antibody (ABclonal Technolog, A2601), and Horseradish peroxidase (HRP)-conjugated goat anti-mouse or anti-rabbit IgG antibodies (DingGuo, Beijing, China).

### Statistical Analysis

Statistical analysis was performed using Graphpad prism 5.0 program (Version 5.2.1350.0 for Windows) and SPSS software (version 19.0). All data were presented as Mean ± SEM unless otherwise indicated. *P*-values were determined by unpaired *t*-test, nonparametric Kruskal-Wallis test between two groups, two-way ANOVA among various treatment groups, and Mann-Whitney-Wilcoxon (*U*-test) in multiple comparisons. Spearman correlation coefficient analysis was used to analyze the correlation between gut microbiota and metabolites using the “ggplot2” and “pheatmap” packages of R software (version 3.3.1). The two-sided, Log-rank (Mantel-Cox) test was used for contrast of mortality curves. All experimental data were representative of three independent experiments with similar results. Statistical significance was determined for ^*^p < 0.05, ^**^p < 0.01, ^***^p < 0.001.

## Results

### The Impact of ESBL-EAEC Infection on Hindgut Microbiota of Dairy Calves

**Figure 1:**
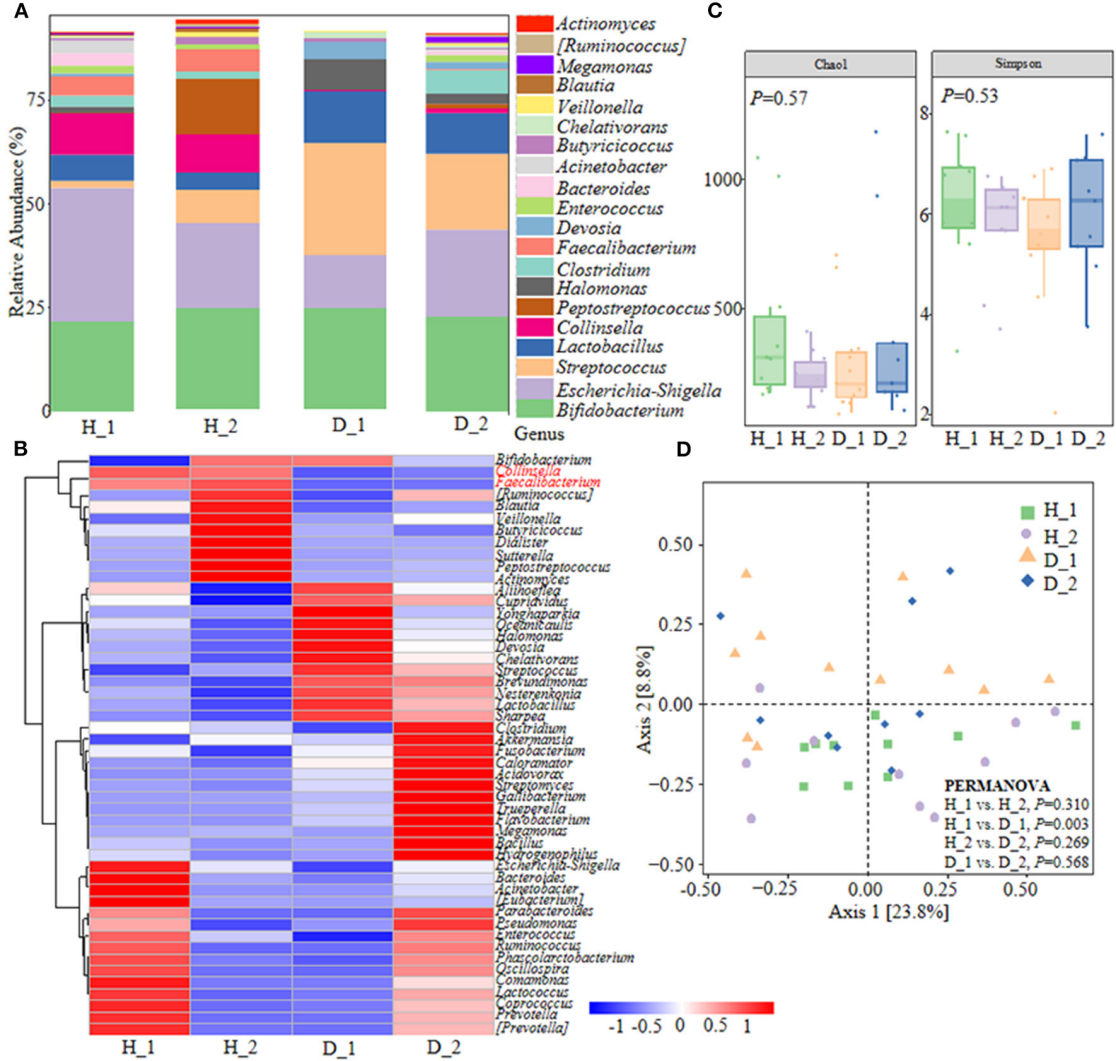
Gut microbiota assembly of neonatal calves post extended-spectrum β-lactamase-producing enteroaggregative *E. coli* (ESBL-EAEC) infection. **(A)** Calf fecal bacterial genera's relative abundance is represented by 99.5% of their community. **(B)** Top 50 bacteria of fecal samples presented using heat map analysis in diarrheal-D or healthy-H calves. The color indicated the relative abundance of the bacteria in the group of samples, and the corresponding relationship between the color gradient and the value was shown in the gradient color block. *Collinsella* and *Faecalibacterium* were labeled red. **(C)** The α-diversity of different groups by Chao1 and Simpson indices. Data were mean ± SEM. *P-*values were determined using the nonparametric Kruskal-Wallis test. **(D)** Principal coordinate analysis (PCoA) of fecal bacteria based on the weighted UniFrac distance matrix. The statistical tests were accomplished using permutational multivariate ANOVA (PERMANOVA) with 999 permutations.

Colibacillus diarrhea closely correlates with the pathogenesis of ESBL-EAEC and is accompanied by the deterioration of antimicrobial resistance in pasture. With the aim of plumbing more about the self-regulatory effects of neonatal calves on ESBL-EAEC-infection control, pooled fecal samples of calves were subjected to 16S ribosomal RNA (rRNA) sequencing analyses to evaluate the diversity and community of fecal microbiota in different groups (Supplementary Table 1). As a result, Firmicutes, Proteobacteria, Actinobacteria, Bacteroidetes, and Fusobacteria were the major phyla in fecal microbiota (Supplementary Figure 1A), with enriched *Coriobacteriaceae, Ruminococcaceae* in healthy groups and *Streptococcaceae, Lactobacillaceae* in diarrheal calves (Supplementary Figure 1B). Among them, considerably abundant *Collinsella* and *Faecalibacterium* were found in both the two healthy groups at the genus level (Figures 1A,B). Then, microbial compositions in groups exhibited a decline trend in D_1 group, as induced by chao1 and Simpson indices (Figure 1C). The overall difference in the microbial structure of diarrheal groups was distinct from healthy ones, according to principal co-ordinates analysis (PCoA) based on weighted UniFrac distance (Figure 1D). The linear discriminant analysis (LDA) effect size (LefSe) algorithm ranked *Collinsella, Coriobacterium, Faecalibacterium, Butyricicoccus*, and *Enterococcus* as the main distinguished bacterial taxa in the H_1 group, while the diarrheal groups were differentiated with *Streptococcus, Peptococcaceae*, and *Gallibacterium*. Similarly, *Clostridia, Collinsella*, and *Coriobacterium* was the main differential bacterial in the H_2 group, while *Halomonas* and *Devosia* in the D_2 group (Supplementary Figures 1C,D). Differentially expressed genes (DEGs) were shown by Kyoto Encyclopedia of Genes and Genomes (KEGG) enrichment pathway analysis and the results implicated some general pathways, including amino acid, carbohydrate, lipid metabolism, biosynthesis of other secondary metabolites, and infectious diseases (Supplementary Figure 2). Briefly, these data demonstrated that ESBL-EAEC-infection was closely related to the alteration of hindgut microbial community structure, especially the temporal changes of microbial biomarker *Collinsella* and *Coriobacterium* in the first 2 weeks of life.

### Intestinal GA as Key Metabolite Mediating Resistance Against Diarrhea Induced by ESBL-EAEC Infection

**Figure 2:**
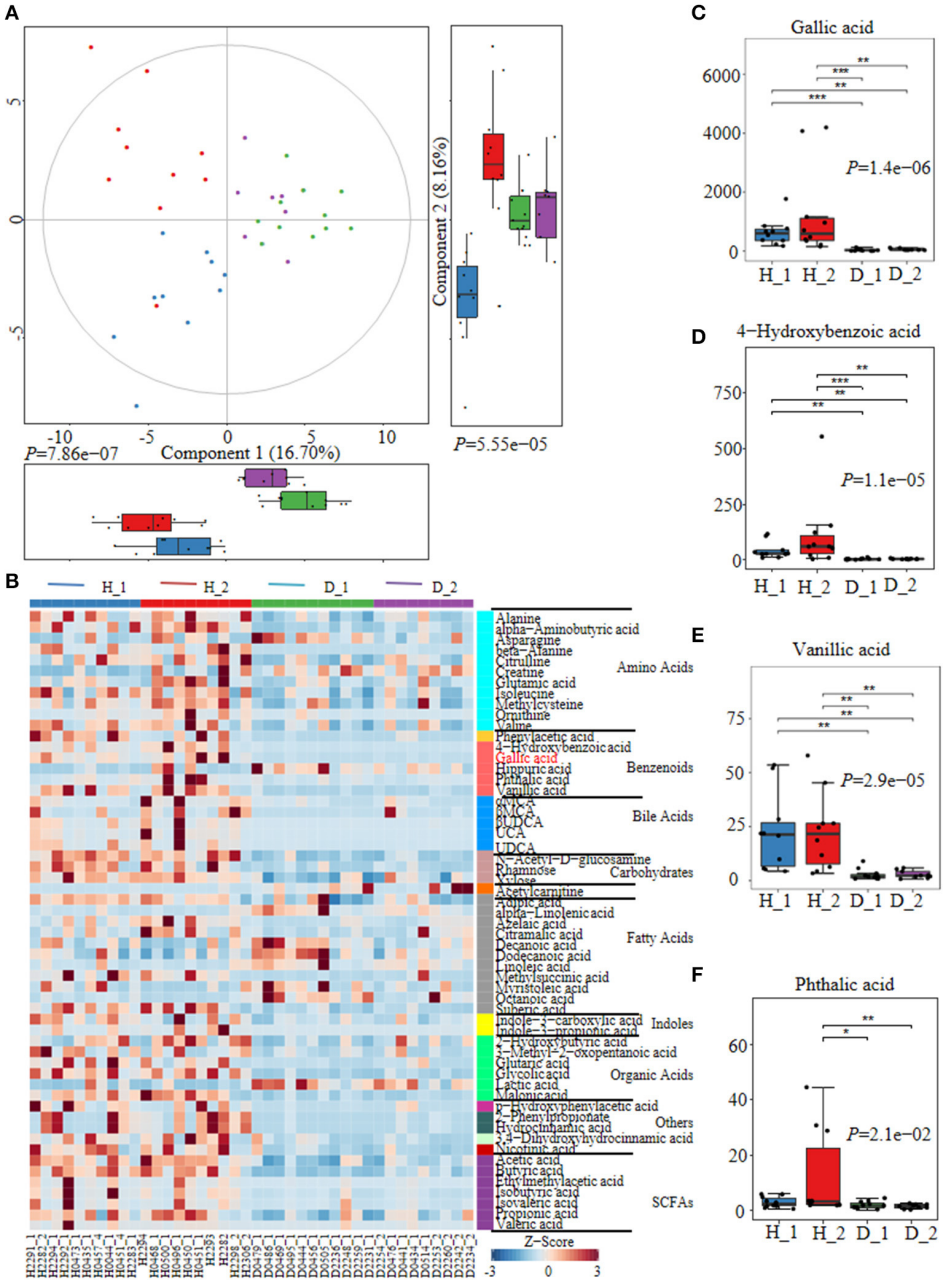
The alterations of fecal metabolome profiles of neonatal calves post ESBL-EAEC infection. **(A)** Partial Least Squares Discriminant Analysis (PLS-DA) was used here to cluster the fecal metabolome profiles of calves. Metabolome profile for the H_1, H_2, D_1, or D_2 groups was shown in the same color, respectively. Data were mean ± SEM. *P*-values were acquired using the nonparametric Kruskal-Wallis test. **(B)** Unweighted paired group method with arithmetic mean (UPGMA) dendrogram was used to cluster the relative abundance of metabolites and results were shown using heatmap analysis. The color indicated the relative abundance of the metabolite in the group of samples, and the corresponding relationship between the color gradient and the value were shown in the gradient color block. The metabolite variation was demonstrated using Z-Score. Gallic acid (GA) was labeled red. The concentrations of fecal GA **(C)**, 4–hydroxybenzoic acid **(D)**, vanillic acid **(E)**, and phthalic acid **(F)** were displayed as box and dot plots. Data were mean ± SEM. *p*-values were determined using the nonparametric Kruskal-Wallis test. *p ≤ 0.05, **p ≤ 0.01, ***p ≤ 0.001.

Fecal metabolites were further detected using untargeted metabolomics to gain a systematic understanding of the interactions among intestinal epithelium, gut microorganisms, and their associated metabolome. Here, fecal metabolites of healthy (*n* = 20) or diarrheal calves (*n* = 20) were analyzed with UPLC-MS/MS system. The comparison of different metabolites revealed a dramatically high relative abundance of benzenoids (2.47 vs.22%), phenols (0.13 vs 0.024%), indoles (0.0090 vs.00084%), SCFA (27.31 vs 13.26%), and amino acids (40.11 vs 23.24%) in healthy calves when compared with diarrheal ones (Supplementary Figure 3A). Similarly, the healthy fecal metabolome separated widely from diarrheal groups based on PLS-DA analysis considering their allocated groups (Component 1, *p* = 7.86e−07; Component 2, *p* = 5.55e−05; Figure 2A, Supplementary Figure 3B). Undeniably, dispersed data points on plots of the metabolome were clearly displayed between H_1 and D_1, H_2 and D_2, H_1 and H_2, and D_1 and D_2 groups (Supplementary Figure 4).

**Figure 3:**
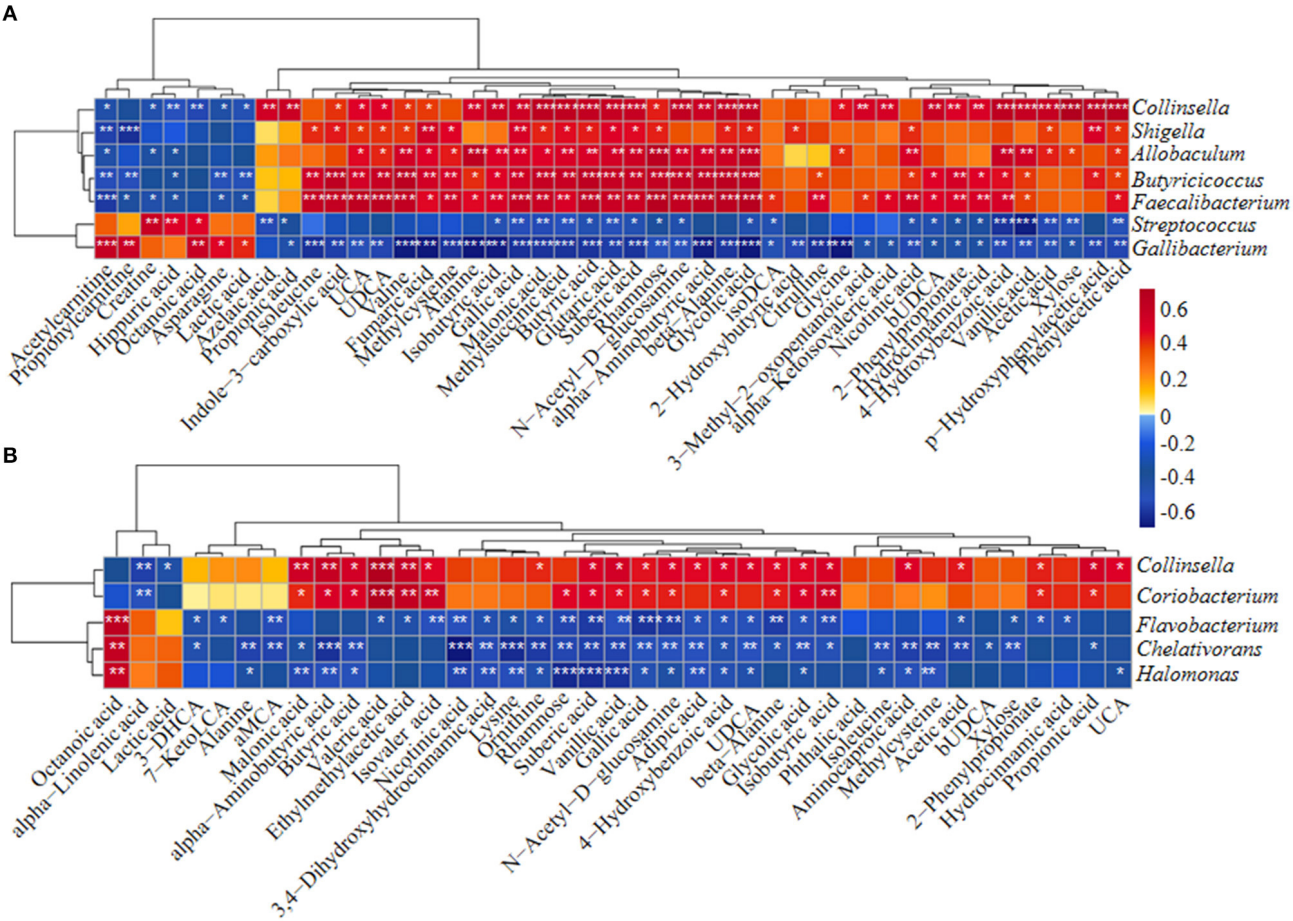
The Spearman correlation between differential gut microbial taxa and their derived metabolites in H_1 vs D_1 **(A)** and H_2 vs D_2 **(B)**. Red squares indicated positive correlation and blue squares indicate negative correlation. The intensity of the color was proportional to the strength of Spearman correlation. *p ≤ 0.05, **p ≤ 0.01, ***p ≤ 0.001.

Differentially abundant fecal metabolites were further identified using an unweighted paired group method with arithmetic mean (UPGMA) dendrogram and appeared in a heatmap, including 57 most diverse metabolites (Figure 2B). Among them, several benzenoid compounds were listed potential biomarkers by a comparison of the healthy and diarrheal calves, including GA (*p* = 1.4e−06), 4-hydroxybenzoic acid (*p* = 1.1e−05), vanillic acid (*p* = 2.9e−05), and phthalic acid (*p* = 2.1e−02) (Figures 2C–F). In addition, relative fold change of quantities and variation significances were presented by volcano plots, as indicated in H_1 vs D_1, H_2 vs D_2, H_1 vs H_2, and D_1 vs D_2 groups (Supplementary Figure 5). According to the markedly altered metabolites, enriched KEGG pathway analyses were involved with valine, leucine, and isoleucine biosynthesis, pantothenate and CoA biosynthesis, propanoate metabolism, linoleic acid metabolism, suggested with alanine, aspartate, and glutamate metabolism (Supplementary Figure 3C). Moreover, as was shown by random forest supervised machine learning algorithm, 10 prominent fecal metabolites contributed to the discrimination power of dairy calf health status, including GA and 4-hydroxybenzoic acid. The relative rank of relative abundance of those 10 metabolites biomarkers was plotted against the healthy status represented by the score of Mean Decrease Gini (Supplementary Figure 3D). Then, the correlation of significantly altered metabolites with specific differentiated microbial taxa was directly revealed. Spearman rank correlation analysis indicated a strong positive correlation between abundant GA and *Collinsella* (R > 0.62, *p* = 0.0026), *Faecalibacterium* (R > 0.67, *p* = 0.00073), *Butyricicoccus* (R > 0.62, *p* = 0.0026), *Enterococcus* (R > 0.47, *p* = 0.027), and *Coriobacterium* (R > 0.47, *p* = 0.029) in the H_1 phase (Figure 3A). Similarly, H_2-enriched GA was closely linked to two hindgut commensals, including *Collinsella* (R > 0.49, *p* = 0.033), and *Coriobacterium* (R > 0.48, *p* = 0.036) (Figure 3B). Notably, the microbes mentioned above were closely associated with enriched 4-hydroxybenzoic acid, vanillic acid, and SCFA, and reduced hippuric acid, octanoic acid, and α-linolenic acid. However, it should be noted that there existed a strong correlation between *Flavobacterium* and octanoic acid (R > 0.70, *p* = 0.00074), *Gallibacterium* and octanoic acid (R > 0.66, *p* = 0.0010), and *Streptococcus* and hippuric acid (R > 0.55, *p* =0.0096), suggesting that hippuric acid, octanoic acid, and linolenic acid could be identified as metabolite biomarkers in those diarrheal calves. Thus, massive cuts in GA and some other benzenoid materials were probably attributed to the decrease of *Collinsella* and *Coriobacterium* abundance observed in healthy calves. These observations indicated that declines of some commensal bacteria reshaped by ESBL-EAEC infection were linked to the impaired biosynthesis of GA or benzenoid pools, further inducing impaired hindgut bacterial structure. Of note, our findings suggested that GA administration could ameliorate intestinal homeostasis by some specific patterns.

### Antibacterial Effects of GA and Suppression of Specific LPS-Induced Colonic Cell Inflammation

**Figure 4:**
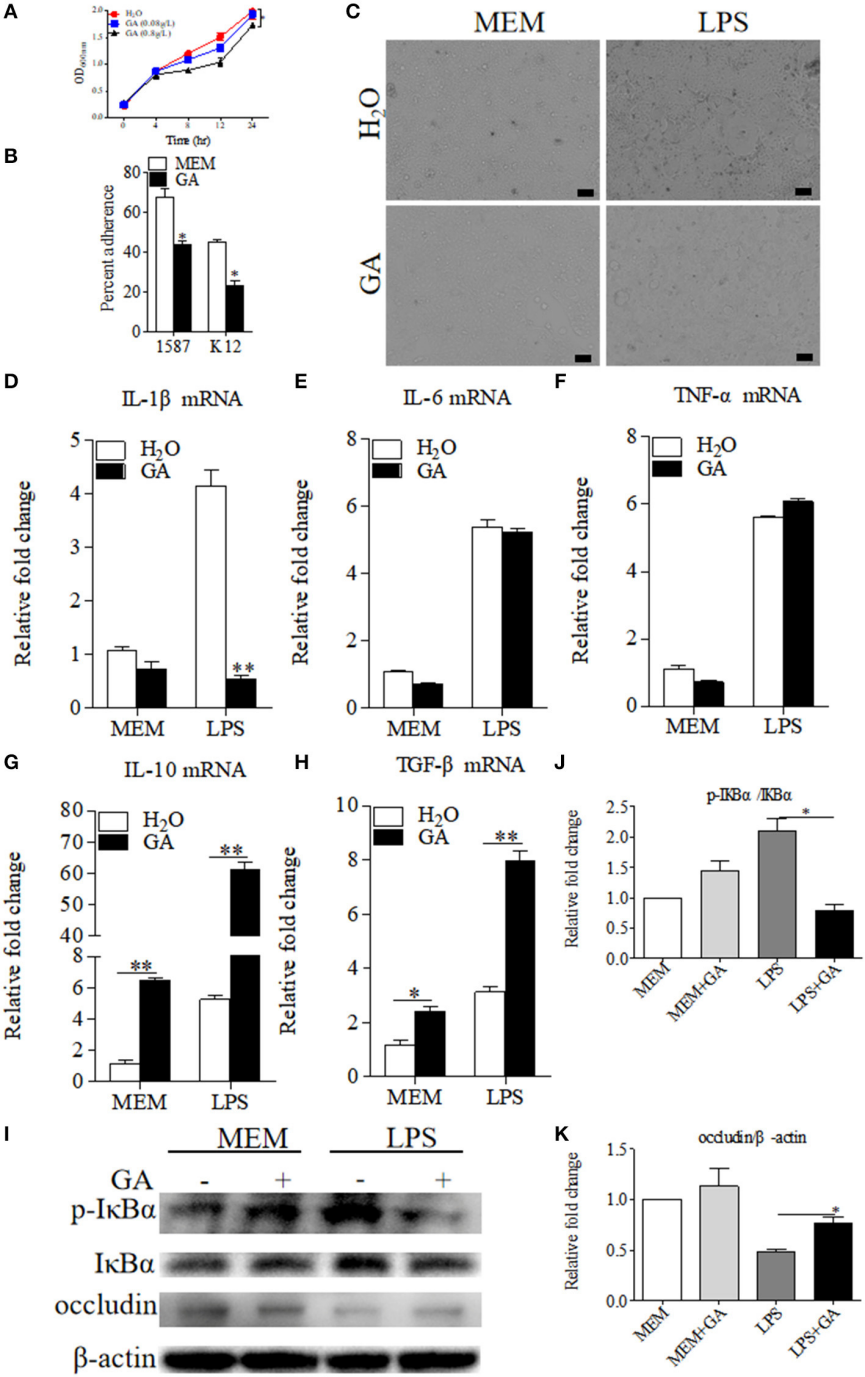
Antibacterial effects of GA on *E. coli* 1587 and suppression of specific LPS-induced inflammation in Caco-2 cells. **(A)** Diarrheagenic *Escherichia coli* (DEC) growth kinetics over a 24 h culture period. Data were shown as means ± SEM from triplicate experiments. Statistical significance was determined using two-way ANOVA. *p ≤ 0.05, **p ≤ 0.01 relative to control group. **(B)** Blockage of GA administration on Caco-2 cell adherence of *E. coli* 1587 and *E. coli* K12. **(C)** Cell morphology transformations of Caco-2 post treatment using lipopolysaccharide (LPS) component extracted from *E. coli* 1587. The relative mRNA expression levels of five representative inflammatory cytokines, IL-1β **(D)**, IL-6 **(E)**, TNF-α **(F)**, IL-10 **(G)**, TGF-β **(H)** in those cells. **(I)** Western blot analyses of the activation of NF-κB pathway and cell tight junction. The relative fold changes of p-IκBα **(J)** and occludin **(K)** were presented as means ± SEM. Statistical significance was analyzed using unpaired *t*-test. *p ≤ 0.05, **p ≤ 0.01. All experimental data were representative of three independent experiments with similar results.

As reported in the literature (12), GA suppressed *E. coli* and viable bacteria biofilm formation and activity *in vitro*. We therefore detected the direct effects of GA on the bacterial growth of clinically relevant ESBL-EAEC strain 1587 *in vitro*. As shown by Figure 4A, ESBL-EAEC growth was markedly inhibited post GA administration at 8, 12, and 24 h in a dose-dependent manner compared to control group. In addition, the 1,587 isolate adhered significantly better than a laboratory *E. coli* K12 strain when adding to human colon adenocarcinoma cell line Caco-2 cell culture. Furthermore, GA addition markedly mitigated cell adherence of both strains, indicating the direct effect of GA to effectively cut off the adhesion of bacteria from the surface of intestinal epithelial cells (Figure 4B).

Considering that LPS was an inseparable component of *E. coli*, the extracted LPS component from ESBL-EAEC was directly added to human colon adenocarcinoma cell line Caco-2 which was pretreated with optimal concentration of GA or sterile water control. As a result, the treatment of GA did not impact cell survival in this specific concentration (data not shown), and cell shrinkage induced by LPS were recovered by GA addition (Figure 4C). To illuminate the underlying mechanism, cells were harvested and were used to quantify the relative mRNA expressions of pro- and anti-inflammatory cytokines. LPS enhanced the expression of IL-1β, IL-6, and TNF-α, and GA pretreatment suppressed the increase of IL-1β (Figures 4D–F), with elevated IL-10 and TGF-β (Figures 4G,H). As TLR4/NF-κB pathway was closely related to bacterial invasion process in intestinal epithelial cells (27), the relative phosphorylation levels of IκBα protein in different treatment groups were further detected. Indeed, GA cotreatment prominently blocked the increased IκBα phosphorylation level induced by LPS (Figures 4I,J). Interestingly, cell integrity analysis showed that GA pretreatment countered the decreased expression level of cell tight-junction related protein occludin (Figures 4I,K). Thus, these results demonstrated that the antibacterial effects of GA administration were mainly mediated by blockage of bacterial growth and cell adherence *in vitro*. GA exposure significantly mitigated NF-κB signaling activation, thus regulating inflammatory cytokines expressions.

### GA Exposure Alleviated ESBL-EAEC-Induced Colitis in Neonatal Mice Peritonitis Sepsis Model

**Figure 5:**
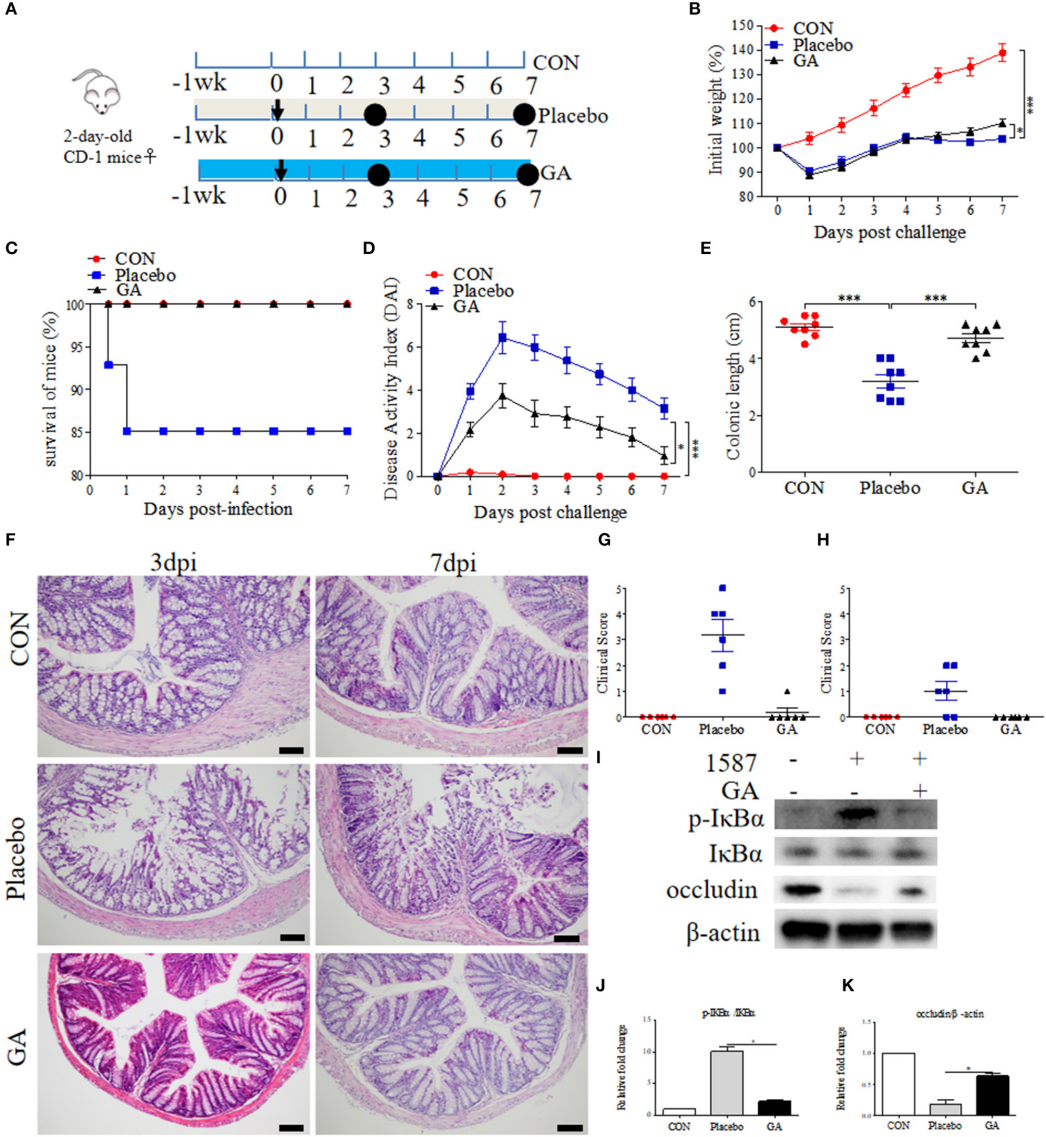
Oral GA improved clinic symptoms and colitis induced by ESBL-EAEC in neonatal mice peritonitis sepsis model. **(A)** Diagram of the mice infection model. Oral sterile water and GA were indicated. The arrow represented infection time point and the circle represented the harvest time. **(B)** Daily body weight changes of mice in this trial. Data were shown as means±SEM (*n* = 12 per group). Statistical significance was determined using two-way ANOVA. *p ≤ 0.05, ***p ≤ 0.001 relative to Placebo group. The survival rate **(C)**, kinetics of daily activity index (DAI) scores **(D)**, and colonic lengths **(E)** of each group in this trial. Survival rate was analyzed using Log-rank (Mantel-Cox) test. H&E-stained colon tissues **(F)**, histological scores of harvested mice colons in 3 dpi **(G)**, 7 dpi **(H)**, and **(I)** Western blot analysis of NF-κB signaling pathway and cell tight junction. The relative fold changes of p-IκBα **(J)** and occludin **(K)** were shown as means ± SEM. Statistical significance was analyzed using unpaired *t*-test. *p ≤ 0.05, ***p ≤ 0.001.

To further study the alleviated effects of GA on ESBL-EAEC infection *in vivo*, 2-day-old mice received daily oral administration of GA for 2 weeks, accompanied by a challenge *via* intraperitoneal injection (Figure 5A). Over that period, clinical symptoms of mice were measured daily. Herein, ESBL-EAEC infection resulted in body weight loss (Figure 5B), high morbidity (Figure 5C), colitis (Figure 5D), reduction in the colonic length (Figure 5E), and histological damage including destruction of glandular structure, deep ulceration of mucosal muscularis, and inflammatory infiltrate (Figure 5F). Notably, GA intervention improved the body weight loss significantly in comparison with Placebo group. The remarkably lower DAI, comprehensive evaluation score of body weight loss, rectal bleeding, stool consistency, and relieved colonic growth inhibition were also displayed. Moreover, GA markedly reversed ESBL-EAEC-induced colonic inflammation and epithelial damage in the blind histological assessment, consisting with largely cut of overall clinical score in both 3 days and 7 days post infection (Figures 5G,H).

Intestinal inflammatory status was closely linked to ESBL-EAEC pathogenesis. We thus detected the pro-inflammatory and anti-inflammatory cytokines production levels in serum and colon tissues. Here, increased serum concentrations of IL-1β were blocked by GA (Supplementary Figures 6A,B). In contrast, reduced IL-10 was recovered to normal status in GA-treated mice (Supplementary Figures 6C,D). Similarly, *E. coli* 1587 infection dramatically stimulated the upregulation of IL-1β, IL-6, IL-10, and TNF-α mRNA transcription levels in mice colons 3 days post infection (Supplementary Figures 6E,G,I,M), but this phenomenon mostly diminished by 7 days post infection except for IL-6 (Supplementary Figures 6F,H,J,N). IL-1β and TNF-α upregulations were significantly reduced by oral GA (Supplementary Figures 6E,M), accompanied by elevated TGF-β mRNA expression level (Supplementary Figure 6L). In addition, oral GA markedly blocked the raised level of IκBα phosphorylation induced by ESBL-EAEC infection (Figures 5I,J), and the suppression of occludin protein expression level was significantly blocked by GA pretreatment (Figures 5I,K).

**Figure 6:**
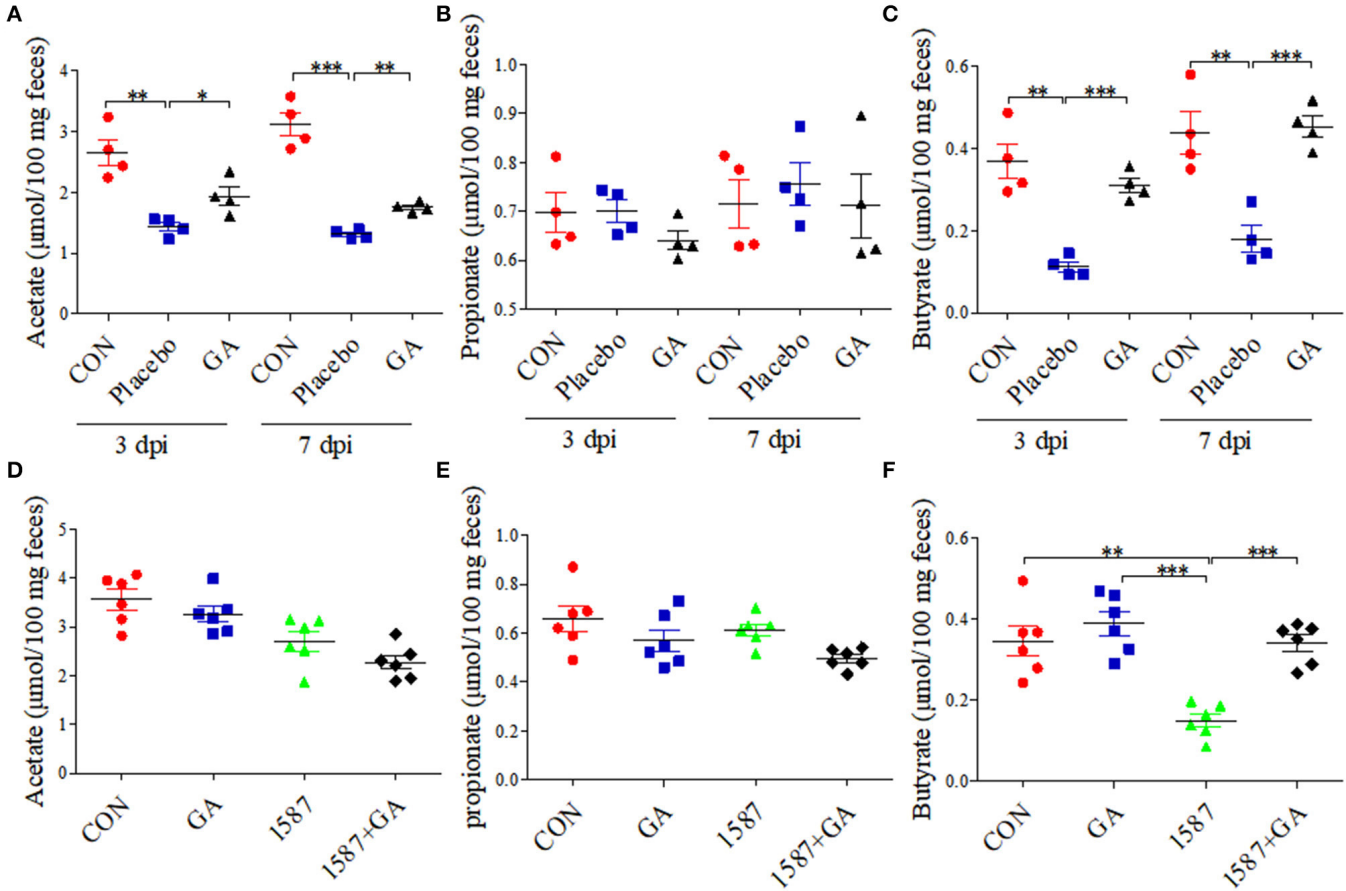
Colonic short-chain fatty acid (SCFA) levels of neonatal mice post oral GA therapy. Concentrations of acetate **(A,D)**, propionate **(B,E)**, and butyrate **(C,F)** upon oral therapy (*n* = 4–6 per group). Data were presented as means±SEM. Statistical significance was analyzed using unpaired *t*-test. ^*^p ≤ 0.05, ^**^p ≤ 0.01, ^***^p ≤ 0.001.

Colon physiology can be mostly affected by active SCFA-producing commensal microorganisms (28). We further evaluated the impact of oral GA on SCFA (acetate, propionate, and butyrate) production in colonic contents using a Gas Chromatography System. Compared with CON group, both acetate and butyrate productions were reduced a lot in the Placebo group except for propionate, but dampened acetate and butyrate recovered to baseline by oral GA in 3 days or 7 days post infection (Figures 6A–C). Importantly, oral GA dramatically suppressed *E. coli* 1587 colonization over the time period (Supplementary Figure 8A). Taken together, mice symptoms and colitis were alleviated by oral GA. Specifically, restored SCFA productions in ESBL-EAEC-infected mice in response to oral GA suggested the reconstruction of hindgut microbial patterns and the beneficial effects were mediated by some commensals in relieving clinic symptoms.

### GA Exposure Alleviated ESBL-EAEC-Induced Colitis in Neonatal Mice Oral Infection Model

**Figure 7:**
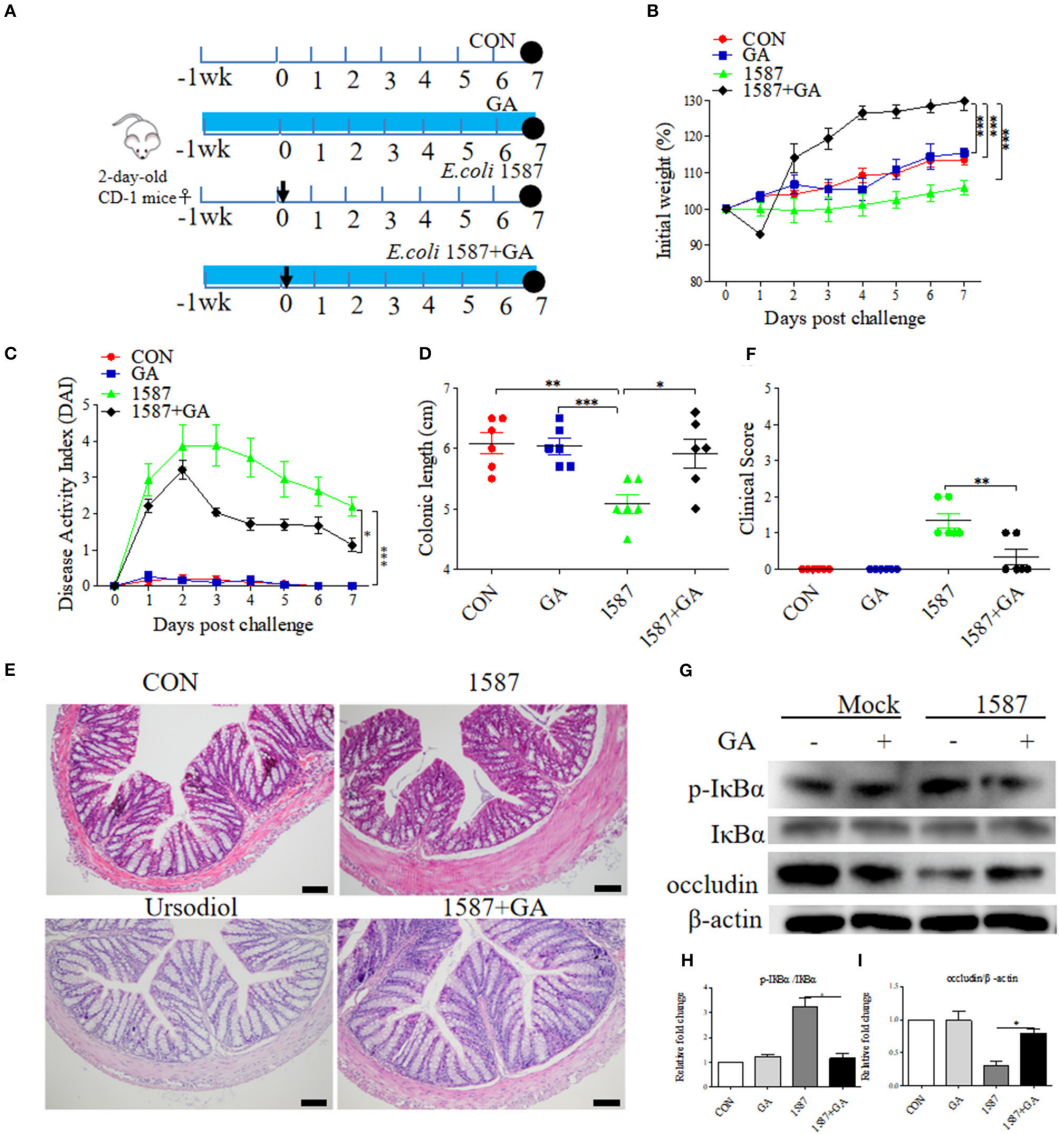
Oral GA alleviated clinic symptoms and colitis induced by ESBL-EAEC in neonatal mice oral infection model. **(A)** Diagram of the mice infection model employed in this study. The arrow represented the time point of infection, and the circle indicated the harvest time. **(B)** Daily body weight changes post treatment. Data were presented as means±SEM (*n* = 6 per group). Statistical significance was determined using two-way ANOVA. *p ≤ 0.05, **p ≤ 0.01, ***p ≤ 0.001 relative to 1587+GA group. **(C)** Kinetics of DAI scores of this trial. **(D)** The colonic lengths of each group. H&E-stained colon tissues **(E)** and histological scores of colons **(F)**. **(G)** Western blot analysis of NF-κB signaling pathway and cell tight junction. The relative fold changes of p-IκBα **(H)** and occludin **(I)** were presented as means±SEM. Statistical significance was analyzed using unpaired *t*-test. *p ≤ 0.05, **p ≤ 0.01, ***p ≤ 0.001.

Considering that infection route was responsible for the virulence and invasion of clinical isolates (29), effects of prophylactic GA supplementation were further explored in neonatal mice oral infection test. As before, 2-day-old mice received daily oral delivery of GA, accompanied by challenge *via* oral gavage of *E. coli* 1587 (Figure 7A). Over that period, detailed records of body weight and disease symptoms of mice were obtained. As a result, ESBL-EAEC infection induced body weight loss (Figure 7B), colitis (Figure 7C), reduced colonic length (Figure 7D), and histological damage consisting of inflammatory cell infiltration, colonic edema, and mucosa damage (Figure 7E). Notably, GA intervention significantly mitigated the body weight loss post infection in comparison with the other groups. The reduced DAI and relieved colonic growth inhibition were also shown. Moreover, oral GA dramatically reversed ESBL-EAEC-induced colonic inflammation and epithelial damage in comparison with 1,587 group, consisting with largely cut overall clinical scores at 7 days post infection (Figure 7F). Of note, oral GA mediated the downregulation of IL-1β expression level in serum (Supplementary Figure 7A) and the upregulation of IL-10 (Supplementary Figure 7B). Similarly, ESBL-EAEC infection elevated transcription levels of IL-1β (Supplementary Figure 7C), IL-6 (Supplementary Figure 7D) and TNF-α (Supplementary Figure 7E) in colonic tissues, but recovered after GA administration with an obvious decline of IL-10 and TGF-β (Supplementary Figures 7F,G). As before, the raised level of IκBα phosphorylation post ESBL-EAEC infection was markedly blocked by GA intervention (Figures 7G,H), with marked upregulation of occludin protein expression level (Figures 7G,I).

Importantly, GA intervention rescued the SCFA concentrations of colonic contents. Results showed that only the concentration of butyrate decreased in the 1,587 group relative to the CON or GA group, but recovered post pretreatment with GA (Figure 6F). In fact, both oral 1,587 infection and GA addition could not affect fecal acetate and propionate productions (Figures 6D,E). As before, oral GA dramatically suppressed 1,587 colonization in colonic tissues (Supplementary Figure 8B).

In summary, the data demonstrated that oral GA could also effectively ameliorate clinic symptoms, colitis, and bacterial colonization inside hindgut of neonatal mice oral infection model. Notably, the enriched butyrate production of ESBL-EAEC-infected mice in response to oral GA administration suggested the improvement of gut microbiota dysbiosis and upregulatory effects of oral GA on SCFAs productions in the hindgut of neonatal animals.

### FMT From GA-Treated Donor Mice Ameliorated Colitis Induced by ESBL-EAEC Infection in Neonatal Mice

**Figure 8:**
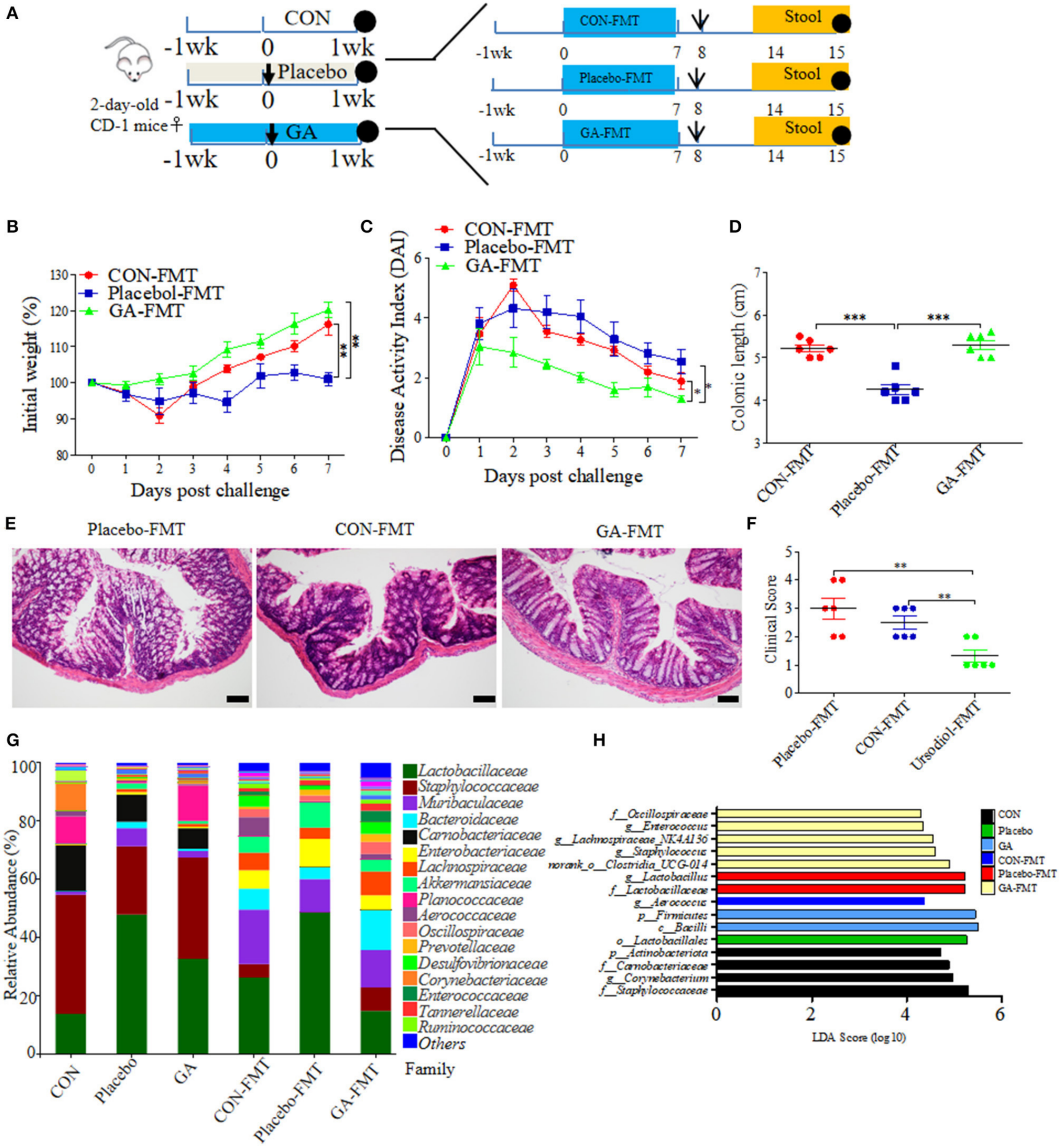
Fecal microbiota transplantation (FMT) of GA-treated mice ameliorated colitis induced by ESBL-EAEC infection. **(A)** Diagram illustrating the neonatal mice model employed in this trial. GA-FMT, CON-FMT, and Placebo-FMT were indicated. The arrow represented infection time point and the circle represented harvest time. Daily body weight changes post infection **(B)** and kinetics of DAI scores in this trial **(C)**. Data were presented as means ± SEM (*n* = 6 per group). Statistical significance was determined using two-way ANOVA. **p ≤ 0.01, ***p ≤ 0.001 relative to Placebo-FMT group. **(D)** Colonic lengths of each group. H&E-stained colon tissues **(E)** and histological scores of colons **(F)**. **(G)** Fecal bacterial family's relative abundance represented by 99.5% of the community. **(H)** Analysis of differences in enriched microbiota taxa shown by linear discriminant analysis (LDA) coupled with effect size measurements (LEfSe) upon FMT.

It has been well studied that gut microbial community patterns contributed a lot to intestinal normal state (30, 31). FMT assays were thus performed in neonatal mice, which were then modeled to colitis by ESBL-EAEC oral infection. Fresh feces were sterilely collected and used for FMT courses, thus allowing for a quantifiable effect of GA-modulated microbiota on protection against syndromes (Figure 8A). As a result, GA-FMT group significantly increased body weight (Figure 8B), attenuated daily DAI (Figure 8C), and lead to recovery of colonic growth inhibition (Figure 8D). Unlike Placebo-FMT group, GA-FMT inoculation dramatically induced recovery of colonic inflammation and epithelial damage (Figure 8E), and largely cut of overall clinical scores (Figure 8F).

A fecal microbiota community was further detected post FMT using 16S rRNA sequencing analysis. As a result, FMT significantly enhanced fecal bacterial community richness of recipients in contrast to their corresponding donor mice. In addition, the α-diversity of GA-FMT markedly raised, as reflected by chao1 or shannon index (Supplementary Figures 9A,B). Specifically, phylogenetic analysis indicated differentiated microbial communities between donor and recipient mice. Phylum-based distinct microbiota profiles were shown by reduced Firmicutes, Actinobacteria, and richer Bacteroidetes in all FMT-treating groups (Supplementary Figure 9C). Diverse microbial compositions were also detected among groups in the family level. A tendency of lower *Lactobacillaceae*, enriched *Bacteroidaceae*, and *Muribaculaceae* were observed in the GA-FMT group compared with Pacebo-FMT or CON-FMT, while obvious increases of *Enterobacteriaceae* were observed in both Placebo and Placebo-FMT groups (Figure 8G). PCoA differentiated the microbiota of those groups based on OTU-level Bray-Curtis dissimilarity matrix, revealing a dispersed data points on plots of donor groups (*R*^2^ = 0.6840, *p* = 0.001) and among recipient groups (*R*^2^ = 0.1402, *p* = 0.296) (Supplementary Figures 9D,E). Notably, the LEfse analysis indicated that oral GA induced enriched *Oscillospiraceae, Enterococcaceae, Lachnospiraceae*, and *Clostridia_UCG-014* and induced abundant *Lactobacillus* in the Placebo or Placebo-FMT group, which were similar to the microbial profiles of healthy calves (Figure 8H). The dominance of various specific members of *Clostridia* correlated with SCFA metabolism among commensal intestinal bacteria (32). Thus, fecal SCFA concentrations of donor and recipient mice were investigated. Unlike propionate, the productions of acetate and butyrate were prominently reduced by *E. coli* 1,587 infection and were then recovered by oral GA (Supplementary Figures 10A–C). Interestingly, GA-FMT markedly upregulated the acetate, propionate, and butyrate productions compared to CON-FMT or Placebo-FMT group (Supplementary Figures 10D–F).

Spearman correlation analysis was further performed to interpret the relevance between differentially enriched bacteria and SCFA productions. *g__norank_f__norank_o__Clostridia_UCG-014* exhibited a strong positive correlation with propionic acid (R > 0.68, *p* = 0.0015), butyric acid (R > 0.63, *p* = 0.0050), *g__Enterococcus* correlated with enriched propionic acid (R > 0.51, *p* = 0.029), abundant acetic acid correlated with *g__Aerococcus* (R > 0.74, *p* = 0.00037), and *g__Bacteroides* (R > 0.61, *p* = 0.00648) (Supplementary Figure 11). In addition, genus *Lachnospiraceae* was associated with increased butyric acid. The data proved the LEfSe results of FMT, an indication of the beneficial effect of GA on SCFA productions of commensal microbes. Not be overlooked was the strong negative correlation between *g__Lactobacillus* and acetic acid (R < −0.59, *p* = 0.0091), propionic acid (R < −0.54, *p* = 0.020), and butyric acid (R < −0.55, *p* = 0.017), which might bring about by enriched lactic acid. This phenomenon was similar to abundant lactic acid and *Lactobacillus* of diarrheal calves in our previous study.

Taken together, clinical symptoms and colitis remission induced by FMT from GA-treated mice could be partly ascribed to hindgut microbial reconstruction of recipients with more abundant *Clostridia_UCG-014, Lachnospiraceae, Oscillospiraceae*, and *Enterococcaceae*, and the recovery of SCFA production, indicating the direct alleviation effects of improved commensal microbiota structure from GA-dosed mice on clinical symptoms and colitis, apart from the direct antibacterial effects on ESBL-EAEC invasion process.

## Discussion

The high prevalence of diarrheagenic *Escherichia coli* (DEC) causes an aggravation of sporadic clinical diarrhea cases, resulting in gastroenteritis outbreak around the world (33). They can be categorized into six pathotypes: enteropathogenic *E. coli* (EPEC), enterohaemorrhagic (Shiga-toxin producing) *E. coli* (EHEC/STEC), enterotoxigenic *E. coli* (ETEC), enteroaggregative *E. coli* (EAEC), enteroinvasive *E. coli* (EIEC), and diffusely adherent *E. coli* (DAEC) (34). Even worse, the pathogenesis of ESBL-EAEC remains unclear, and antibiotic abuse accelerates the rapid spread of MDR, posing a severe threat to public health (35).

Multi-drug resistant ESBL-EAEC infection correlates with extensive clinical diarrheal cases among animals and humans, especially the rising occurrence of extended-spectrum beta lactamase (ESBL)-producing isolates (36). Here, we aimed to attenuate the suffering post ESBL-EAEC infection among neonatal calves by investigating specific antibiotic alternative or synergist with the aim of blocking further resistance dissemination. Thus, we directly isolated clinical ESBL-EAEC from neonatal calves in conventional pasture, which had been proved to harbor MDR genes and enterotoxin EAST1. Of note, ESBL-EAEC strains with highly-expressed EAST1 have been proved to result in diarrhea principally in humans, and then set up bacterial rapid adaption and propagation among calves, piglets, and the other animals (37). For the most part, ESBL-EAEC infections are asymptomatic carriage and self-limiting among host, but some external factors, such as colostrum, diets, and environmental microorganism community, correlate closely to the development and progression of calf diarrhea (1). Currently, we only separated EAEC strains due to geographical restrictions and limited large ranch opening permissions. So, the data could only reveal the antibacterial effects of GA on ESBL-EAEC.

We will surely proceed the further investigations of the effects of GA on the pathogenesis of other DEC strains thoroughly in our subsequent clinical research, along with the isolations of these stains from calves of other conventional pastures in the other parts of China. This is to systematically clarify whether GA provided superiorly antibiotic alternative for early intervention of improving young ruminant healthcare with reduced DEC transmission.

Diet composition correlates the alterations of gut microbial diversity in those young ruminants (38). Herein, neonatal calves of this trial were only fed milk replacer (same intake of the same age), and neonatal mice were fed with breast milk of littermate female mice, thus detecting the specific host regulators that affected the occurrence and progression of ESBL-EAEC infection. To illustrate how did ESBL-EAEC affect gut microbiota accurately, the bacterial compositions among H_1, H_2, D_1, and D_2 groups were compared by time periods. Of interest, diarrhea cases mostly occurred in newborn calves between 4 to 11 days of age during our 3-week time-period study. Although the relative abundance of total bacterial genus showed no obvious difference among groups, and the shannon or simpson index was similar in both H_1 vs H_2 and D_1 vs D_2 phases, but distinct gut microbiome structures were detected. Importantly, gut commensal bacteria are closely linked to host nutrient acquisition, immune recognition, and pathogen exclusion, thus acting as a ligament between endogenous and exogenous factors (39, 40). Here, our observations demonstrated that ESBL-EAEC infection promoted the diarrhetic process by altering the compositions of gut microbiota, including a severe reduction in the abundance of *Coriobacteriaceae* and *Ruminococcaceae*, which could be deemed the indicator phylotypes of active intestinal tract as revealed by LEfse analysis of H_1 vs D_1 and H_2 vs D_2 groups. Consistently, similar change in gut microbiota has been validated in inflammatory bowel diseases (IBD) patients (41). Meanwhile, diarrheal calves were associated with dominant *Streptococcus* and *Halomonas* in D_1 and D_2 respectively. Notably, the hindgut microbial was dominated by lactic acid bacteria, including *Lactobacillus, Streptococcus*, and *Bifidobacterium*, as indicated by abundant lactic acid in diarrheal groups, which were attributed to the prominent position of hindgut fermentation in metabolizable energy supply during the 1 weeks after birth (42).

Plunged commensal bacteria *Collinsella* and *Coriobacterium* strongly correlated with the reduced GA over the whole time period. However, the inherent influential mechanisms of these two bacteria on GA production remained elusive. Importantly, our results revealed higher prevalence of SCFA-producing bacteria belonging to *Ruminococcaceae* (containing *Faecalibacterium* and *Butyricicoccus*) in healthy calves (43–46), with higher concentrations of acetic acid, propionate acid, butyric acid, isobutyric acid, valeric acid, and isovaleric acid in healthy groups. Thus, the beneficial effects of SCFA on the gut mucosal immune responses also provided adjunctive effects on the fight against ESBL-EAEC infection (47, 48). Future studies are warranted to reveal the specific microbiological mechanism behind GA metabolisms, and synergism of GA and SCFA regeneration in anti-bacterial effects.

It is well-known that polyphenols are major sources of natural health products which are conducive to human healthcare (49, 50). As an active phenolic acid in dietary polyphenols, GA (trihydroxybenzoic acid), is a widely distributed secondary metabolite present in various vegetables, fruits, and herbal medicines (6). In addition, GA, 4-hydroxybenzoic acid, vanillic acid, protocatechuic acid, and syringic acid are hydroxybenzoic derivatives. Unfortunately, just like the other polyphenol components, GA and its derivatives can be quickly absorbed in gut lumen, accompanied with rapid metabolism and a high elimination rate *in vivo*, thus inducing poor oral bioactivity (51). Thus, development of sustained or controlled-release oral dosage form for GA is urgently needed. Over the past few years, limited evidence indicated that GA markedly improved ulcerative colitis (UC) *via* affecting gut microbiota composition and their derived metabolites in mice (52). On the other hand, GA has been related with worsen disease in colorectal cancer. GA administration specifically impaired the ability of mutant p53 to suppress hyperactivation of Wingless-type MMTV integration site family (WNT) pathway and continuous presence of GA was required to prevent the tumor-suppressive property of mutant p53 in jejunal organoids, which opened up possible preventive and therapeutic options for cancer (53). However, the exact molecular mechanism of how treatment with GA overrides the WNT-blocking effect of mutant p53 remains unclear. Importantly, GA has potent antimicrobial activities against *Escherichia-Shigella, Eumycetes*, and influenza A virus infections (12, 54, 55). However, there is no adequate evidence to confirm the effectiveness of GA in ESBL-EAEC infection. Here, GA intervention suppressed ESBL-EAEC growth in a dose-dependent manner *in vitro*, which was similar to previous publications. Fecal GA was significantly enriched in the two stages of healthy calves, indicating its lasting protective effect on the occurrence and progression of those infection cases in their early lives. Actually, GA synthesis process is a typical enzymatic reaction, mediated by various gut microbes. Tannase is a key microbial enzyme involved in GA synthesis from gallotannins. Likewise, microbial gallate decarboxylase induces the transformation from GA to pyrogallol (56). Herein, according to our KEGG pathway analyses following 16S rRNA sequencing, we detected a relative higher abundance of bacteria mediating benzoate degradation in both D_1 (D_1 vs H_1, log_2_FC = −0.2414) and D_2 (D_2 vs H_2, log_2_FC = −0.1585) groups (data not shown). Thus, we speculated that GA to pyrogallol was probably involved in the benzoate degradation process. Meanwhile, further transcriptome data were necessary for detecting whether the genes (tannase/gallate decarboxylase) harbored by some bacterial species were overrepresented or underrepresented in this analysis. Of note, abundant phenolic acids, including 4-hydroxybenzoic acid, vanillic acid, and phthalic acid, had also been displayed in fecal metabolome of healthy calves, attributing to the same origin of hydroxybenzoic metabolism and their synergistic effects against infection. Beyond that, some specific bile acids, SCFA, indoles, and unabsorbed carbohydrates were also massed in healthy groups, including ursodeoxycholic acid (UDCA), acetic acid, butyric acid, rhamnose, and indole-3-propionic acid. Among them, UDCA, a natural secondary bile acid derived from gut microbiota, is discovered to possess an excellent effect of colonic epithelial cell protection against oxidative damage and cell apoptosis (57). Rhamnose, an important ingredient of suface-associated exopolysaccharide (EPS) in many probiotics, can mediate displacement of pathogenic organisms through the competitive occupancy of adhesion sites and stimulation of the immune system (58, 59). Indoles belong to gut microbiota-derived trypotophan metabolites, which can effectively suppress inflammatory responses (60). Indeed, apart from GA and SCFA metabolisms, those aforementioned metabolites could also display probiotic properties against ESBL-EAEC infection. In other words, GA and gut microbiota have mutual effects: gut microbiota can mediate GA metabolism, and GA induces microbiota to a more favorable composition and activity, including the production of SCFA in the colon. Thus, we will carry on our study to detect whether enriched GA consequence of the microbiota composition using feces (from healthy and diarrheal calves) and gallotannins or benzoic acid *in vitro*. Unfortunately, abundant linoleic acid and hippuric acid were much concerned here. Linoleic acid, dietary polyunsaturated fatty acids (PUFAs), can serve as key biomarkers for the progression of UC and gut microbiota dysbiosis, and destroy the cell membrane of some probiotics (61, 62), which is consistent with enriched linoleic acid metabolism pathway in our KEGG analysis. While, concentrated hippuric acid, a protein-bound uremic toxin, had been closely linked to the upregulation of pro-inflammatory cytokines and oxidative stress, which could accelerate the deterioration of disease and indicated its utility in calf feces as a plausible hallmark of frailty post ESBL-EAEC infection among neonatal calves (63).

To investigate the alleviated effects of GA on clinical symptoms and colitis, GA was added to Caco-2 cell culture directly. GA exposure maintained cell integrity and barrier function, and suppressed the transcription levels of IL-1β, with an upregulation of IL-10 and TGF-β expressions post stimulation of ESBL-EAEC-LPS. Then, oral delivery of GA was conducted in neonatal mice peritonitis sepsis or oral infection model, and therapeutic administration of GA ameliorated clinic symptoms, as shown by reduced mortality, DAI, body weight loss, and histological damage of mice. Actually, one familiar innate immune pathway involved in LPS-mediated activation in intestinal epithelial cells belongs to the NF-κB signaling pathway, which induces the transcription of pro-inflammatory cytokines, including TNF-α, IL-1β, and IL-6 (64). Similar to previous report (14), GA pretreatment induced repression of IκBα phosphorylation and enhanced tight junction. More importantly, GA reduced the nuclear accumulation of p-STAT3 and degradation of IκBα, as indicated by a significant reduction in colonic pro-inflammatory cytokines in dextran sodium sulfate (DSS)-exposed mice (14). Collectively, previous and our data suggested that GA exerts potentially clinically useful anti-inflammatory effects through the suppression of NF-κB pathway and IL-6/p-STAT3 activation.

Importantly, different animals have completely different digest system, gut microbiome, number and profile of immune cells following development or aging-process. Here, we just detected the anti-ESBL-EAEC effects and colitis alleviation of GA on neonatal mice. While, we will carry on this research using neonatal calf infection model, thus detecting the exact molecular mechanisms of GA on amelioration of hindgut bacterial disturbance, long-term effect on gut microbes maturation, colitis attenuation, T-cell maturation of lamina propria, and SCFAs productions in digesta, thus accelerating the application of GA in livestock industry.

As a highly effective therapy treating *Clostridioides difficile* infection (CDI), inflammatory bowel disease (IBD), and irritable bowel syndrome (IBS) (65, 66), FMT has been applied to both monogastric animals (67, 68) and ruminants (69). Similarly, to previous FMT trials, the beneficial effects of GA-FMT were further detected in the neonatal mice. Interestingly, GA-FMT ameliorated clinical symptoms and colitis, which was in consistent with the prominent effects of oral GA. Importantly, GA-FMT displayed better alleviated symptoms than CON-FMT, suggesting that GA-mediated hindgut microbiome alteration mattered a lot during ESBL-EAEC infection. Here, GA administration had little impact on bacterial diversity, but dramatically decreased the relative abundances of Bacteroidetes and Actinobacteria in neonatal mice. Meanwhile, significant increases of fecal acetate and butyrate productions post GA treatment. This could be caused by GA-induced fecal metabolite alterations, or changes in metabolites correlates to GA intake, which was similar to a previous study of animal model on the attenuation of DSS-induced rat UC by GA (52). Therefore, GA could effectively promote the gut microbiota fermentation of polysaccharides. Moreover, the nuclear magnetic resonance testing data also reveals that GA-induced upregulations of bile acid metabolism and carbohydrate metabolism, and significant decline in amino acid metabolism, which was in consistent with our results (14). FMT had significant accelerative effects on bacterial community richness. GA-FMT greatly influenced the microbial structure compared with donors, which could be attributed to two reasons: one was the existence of undigested GA in fecal excretion of mice donors, thus continuously affecting gut microbiota composition of recipients and dramatically suppressing the growth of *Enterobacteriaceae*; the other reason was the probiotic characteristics of fecal bacteria and changed fecal metabolites, considering that GA-FMT dramatically upregulated *Clostridia_UCG-014, Lachnospiraceae, Oscillospiraceae*, and *Enterococcaceae*, with enriched SCFA production. On the other hand, the transference of some microbiota post FMT was apparent, including the transplantations of *Lactobacillaceae* from Placebo to Placebo-FMT groups, and Firmicutes from GA to GA-FMT groups as revealed by LEfSe results. It could be due to more efficient transplantations of those bacteria even in the presence of GA. Remarkable increases of fecal acetate, propionate, and butyrate productions post GA-FMT suggested the beneficial effects of GA-FMT on the improvement of hindgut microbiota structure and enrichment of SCFA-producing bacteria in hindgut. The above results suggested that GA-FMT had similar effects as GA itself, underlining the improved hindgut microbiota had medicinal benefits during ESBL-EAEC infection state. FMT therapy showed great potential among the most colibacillus diarrhea therapeutics in the coming future. Based on above empirical results, we concluded that the direct blockage of *E. coli* growth and invasion, improvement of hindgut microbiota structure, and SCFA generation recovery by GA intervention were the key reasons accounting for the alleviated effects on gut microbiota dysbiosis and colitis.

Collectively, multi-omics analyses of fecal samples of growing neonatal calves revealed the differences of the hindgut microbiota and fecal metabolites. ESBL-EAEC infection mainly influenced the gut microbiota structure, particularly for the collapsed commensal bacteria *Collinsella* and *Coriobacterium*, accompanied by a sharp reduction of GA, reduced SCFA, and some other critical prebiotics in fecal metabolome. GA exposure was evidenced to block bacterial growth and invasion processes, ameliorating hindgut microbiota dysbiosis, clinical symptoms, and colonic inflammation induced by ESBL-EAEC infections in neonatal animals. These changes were believed to be achieved by reconstruction of commensal colonization and upregulation of SCFA productions, mainly through aggregated *Clostridia_UCG-014, Lachnospiraceae, Oscillospiraceae*, and *Enterococcaceae*. These findings provided innovative insights into the GA-mediated attenuation of ESBL-EAEC infections among young ruminants and would facilitate the reduction of ESBL-EAEC transmission and antimicrobial usage.

## Data Availability Statement

The datasets presented in this study can be found in online repositories. The names of the repository/repositories and accession number(s) can be found in the article/Supplementary Material.

## Ethics Statement

The animal study was reviewed and approved by the Institutional Ethics Committees of China Agricultural University.

## Author Contributions

ZC, ZH, YM, YW, WW, HY, and SheL designed the experiments. YM and ZH conducted the experiments and analyzed the data. YM, ShuL, XC, and JX collected and performed the analysis of samples. ZC and ZH wrote the manuscript. All authors have read and approved the final manuscript.

## Funding

The research was supported by grants from the National Key Research and Development Program of China, grant number: 2021YFF1000703-03.

## Conflict of Interest

The authors declare that the research was conducted in the absence of any commercial or financial relationships that could be construed as a potential conflict of interest.

## Publisher's Note

All claims expressed in this article are solely those of the authors and do not necessarily represent those of their affiliated organizations, or those of the publisher, the editors and the reviewers. Any product that may be evaluated in this article, or claim that may be made by its manufacturer, is not guaranteed or endorsed by the publisher.

## Supplementary Material

Supplementary Figure 1 Gut microbiota assembly of neonatal calves post extended-spectrum β-lactamase-producing enteroaggregative *E. coli* (ESBL-EAEC) infection. The relative abundance of fecal bacterial phylum **(A)** and family **(B)** represented by 99.5% of the community. The enriched gut microbiota taxa were shown by linear discriminant analysis (LDA) coupled with effect size measurements (LEfSe) of H_1 vs D_1 **(C)** and H_2 vs D_2 **(D)**.

Supplementary Figure 2 Kyoto Encyclopedia of Genes and Genomes (KEGG) analysis of differentially expressed genes (DEGs) belonging to H_1 vs D_1 **(A)** and H_2 vs D_2 **(B)**. Respective name of each KEGG pathway was shown on the left, and the pathway categories were indicated on the right.

Supplementary Figure 3 Alterations of fecal metabolome profiles of neonatal calves post ESBL-EAEC infection. **(A)** The classifications of total metabolome compounds in H_1, H_2, D_1, and D_2 groups. The relative abundances of metabolites in different groups and the corresponding significant changes were shown. *P* values were determined using the nonparametric Kruskal-Wallis test. ^*^*p* ≤ 0.05, ^**^*p* ≤ 0.01, ^***^*p* ≤ 0.001. **(B)** Three-dimensional Partial Least Squares Discriminant Analysis (PLS-DA) was used here to cluster the fecal metabolome profiles of calves. The metabolome profiles for the H_1, H_2, D_1, or D_2 group was shown in the same color, respectively. **(C)** KEGG pathway enrichment analysis associated with the dramatically changed metabolites. Respective name of KEGG pathway was shown on the left, and the corresponding *P* value was shown on the right with a gradient color. *P* values were acquired following two-side Fisher's exact tests with Benjamini-Hochberg correction for multiple testing. **(D)** Differentiated metabolites were displayed using random forest supervised machine learning algorithm among H_1, H_2, D_1, and D_2 groups. Respective name of metabolite was shown on the left. Top 10 metabolites in fecal samples were shown with different colors, and the rank values are shown as Mean Decrease Gini.

Supplementary Figure 4 Fecal samples cognate metabolomics analyses in healthy and diarrheic calves. PLS-DA for neonatal calves in H_1 vs D_1 **(A)**, H_2 vs D_2 **(B)**, H_1 vs H_2 **(C)**, and D_1 vs D_2 **(D)**. H, healthy calves; D, diarrheic calves.

Supplementary Figure 5 Differentiated fecal metabolomics analyses from healthy and diarrheic calves representing by volcano maps. Enriched metabolites were identified by analyses with Fold Change (FC) values ≥1 (|log2FC|≥0) and *P* values of < 0.05. Significant upregulation and downregulation of metabolites were shown separately in red and blue. Metabolites with no obvious changes (Noise) were displayed in gray. The distinguished metabolites of H_1 vs D_1 **(A)**, H_2 vs D_2 **(B)**, H_1 vs H_2 **(C)**, and D_1 vs D_2 **(D)** were respectively shown. Each counting numbers of upregulated or downregulated metabolites were shown on the right.

Supplementary Figure 6 Relative expression levels of inflammatory cytokines in neonatal mice peritonitis sepsis model. Serum levels of IL-1β **(A,B)** and IL-10 **(C,D)** assessed by ELISA. Colonic mRNA levels of five representative cytokines, namely, IL-1β **(E,F)**, IL-6 **(G,H)**, IL-10 **(I,J)**, TGF-β **(K,L)**, and TNF-α **(M,N)**, assessed by qRT-PCR. Data were presented as means ± SEM. Statistical significance was analyzed using unpaired *t*-test. ^*^*p* ≤ 0.05, ^**^*p* ≤ 0.01, ^***^*p* ≤ 0.001.

Supplementary Figure 7 Relative expression levels of inflammatory cytokines in neonatal mice oral infection model. Serum levels of IL-1β **(A)** and IL-10 **(B)** assessed by ELISA. Colonic mRNA expression levels of five representative cytokines, namely, IL-1β **(C)**, IL-6 **(D)**, TNF-α **(E)**, IL-10 **(F)**, and TGF-β **(G)**, assessed by qRT-PCR. Data were presented as means ± SEM. Statistical significance was analyzed using unpaired *t*-test. ^*^*p* ≤ 0.05, ^**^*p* ≤ 0.01, ^***^*p* ≤ 0.001.

Supplementary Figure 8 The suppressive effect of oral gallic acid (GA) on *E. coli* 1587 colonization in neonatal mice sepsis **(A)** and oral infection **(B)** models. Data were presented as means ± SEM. Statistical significance was analyzed using unpaired t-test. ^*^*p* ≤ 0.05, ^**^*p* ≤ 0.01, ^***^*p* ≤ 0.001.

Supplementary Figure 9 Fecal microbiota transplantation (FMT) of oral GA mice reconstructed the hindgut microbiota in neonatal mice post infection. The α-diversity of different groups by chao1 **(A)** or shannon index **(B)**. Data were shown as means ± SEM. Statistical significance was analyzed using unpaired *t*-test. ^*^*p* ≤ 0.05, ^**^*p* ≤ 0.01, ^***^*p* ≤ 0.001. **(C)** Mice fecal bacterial phylum's relative abundance represented by 99.5% of their community. Principal coordinate analysis (PCoA) of fecal bacteria based on the weighted UniFrac distance matrix of FMT donors **(D)** and recipients **(E)**. The data were assessed using permutational ANOVA (PERMANOVA) analysis with 999 permutations.

Supplementary Figure 10 Colonic short-chain fatty acid (SCFA) levels of neonatal mice donors and recipients. The concentrations of acetate **(A)**, propionate **(B)**, and butyrate **(C)** in donors upon oral GA therapy (*n* = 4 per group). Concentrations of acetate **(D)**, propionate **(E)**, and butyrate **(F)** in recipients upon FMT (*n* = 6 per group). Data were shown as means ± SEM. Statistical significance was analyzed using unpaired *t*-test. ^*^*p* ≤ 0.05, ^**^*p* ≤ 0.01, ^***^*p* ≤ 0.001.

Supplementary Figure 11 Spearman correlation between fecal microbiota of FMT recipients and SCFA productions, including acetic acid, propionic acid, and butyric acid. The red color denoted a positive correlation, while blue color denoted a negative correlation. The intensity of the color was proportional to the strength of Spearman correlation. ^*^*p* ≤ 0.05, ^**^*p* ≤ 0.01, ^***^*p* ≤ 0.001.

Supplementary Table 1 Genomic analysis of DEC bacterial strain.

Supplementary Table 2 Sampling information of calves used in the current study.
