# Supplementary figures and images for: Protective Effects of Intestinal Gallic Acid in Neonatal Dairy Calves Against Extended-Spectrum β-lactamase Producing Enteroaggregative Escherichia coli Infection: Modulating Intestinal Homeostasis and Colitis
Source: Front Nutr. 2022 Mar 24;9:864080. doi: 10.3389/fnut.2022.864080 (PMC8988045; doi:10.3389/fnut.2022.864080)

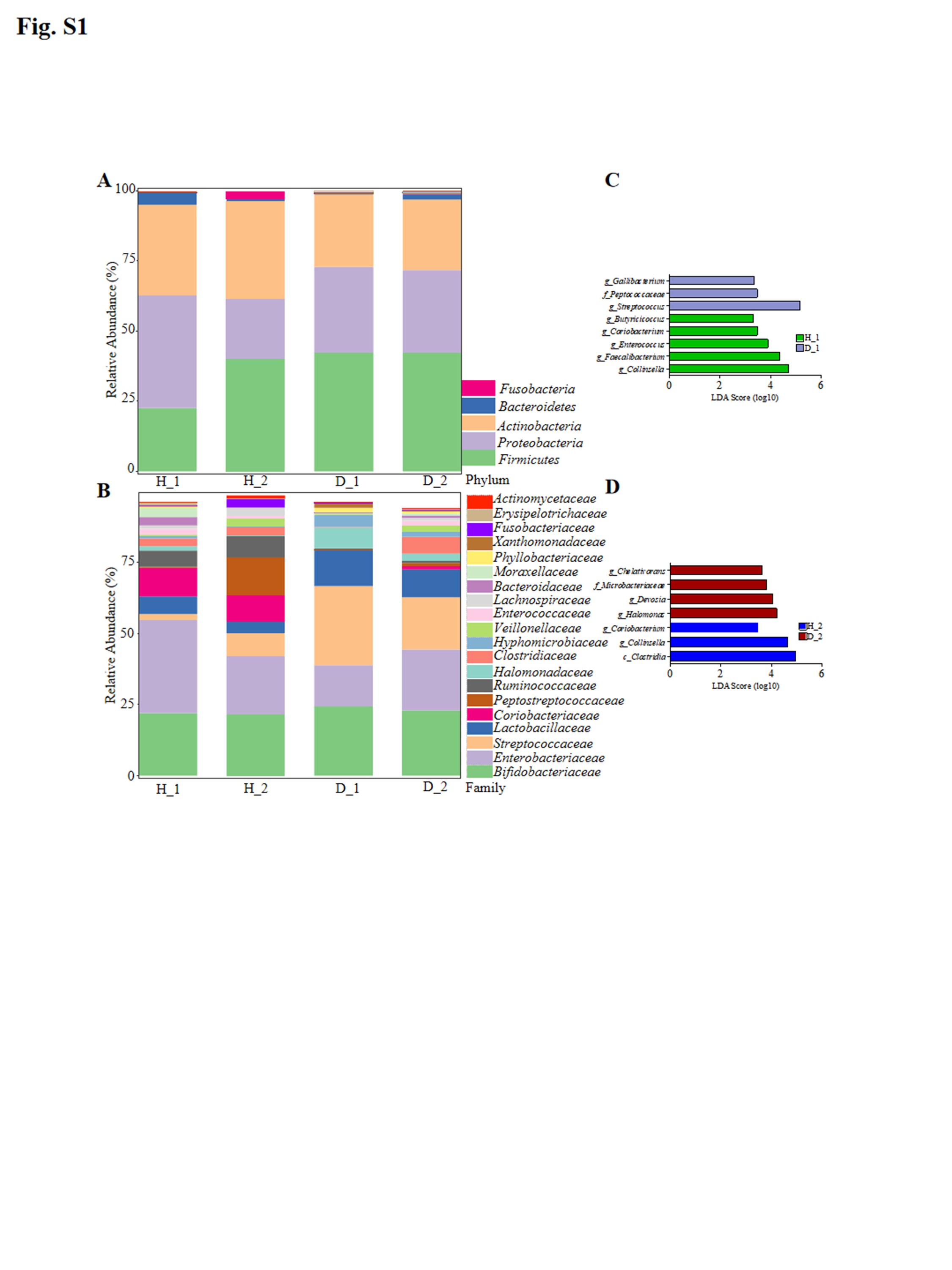

Supplement: Supplementary Figure 1 — Gut microbiota assembly of neonatal calves post extended-spectrum β-lactamase-producing enteroaggregative E. coli (ESBL-EAEC) infection. The relative abundance of fecal bacterial phylum (A) and family (B) represented by 99.5% of the community. The enriched gut microbiota taxa were shown by linear discriminant analysis (LDA) coupled with effect size measurements (LEfSe) of H_1 vs D_1 (C) and H_2 vs D_2 (D). [file Image_1.TIF]

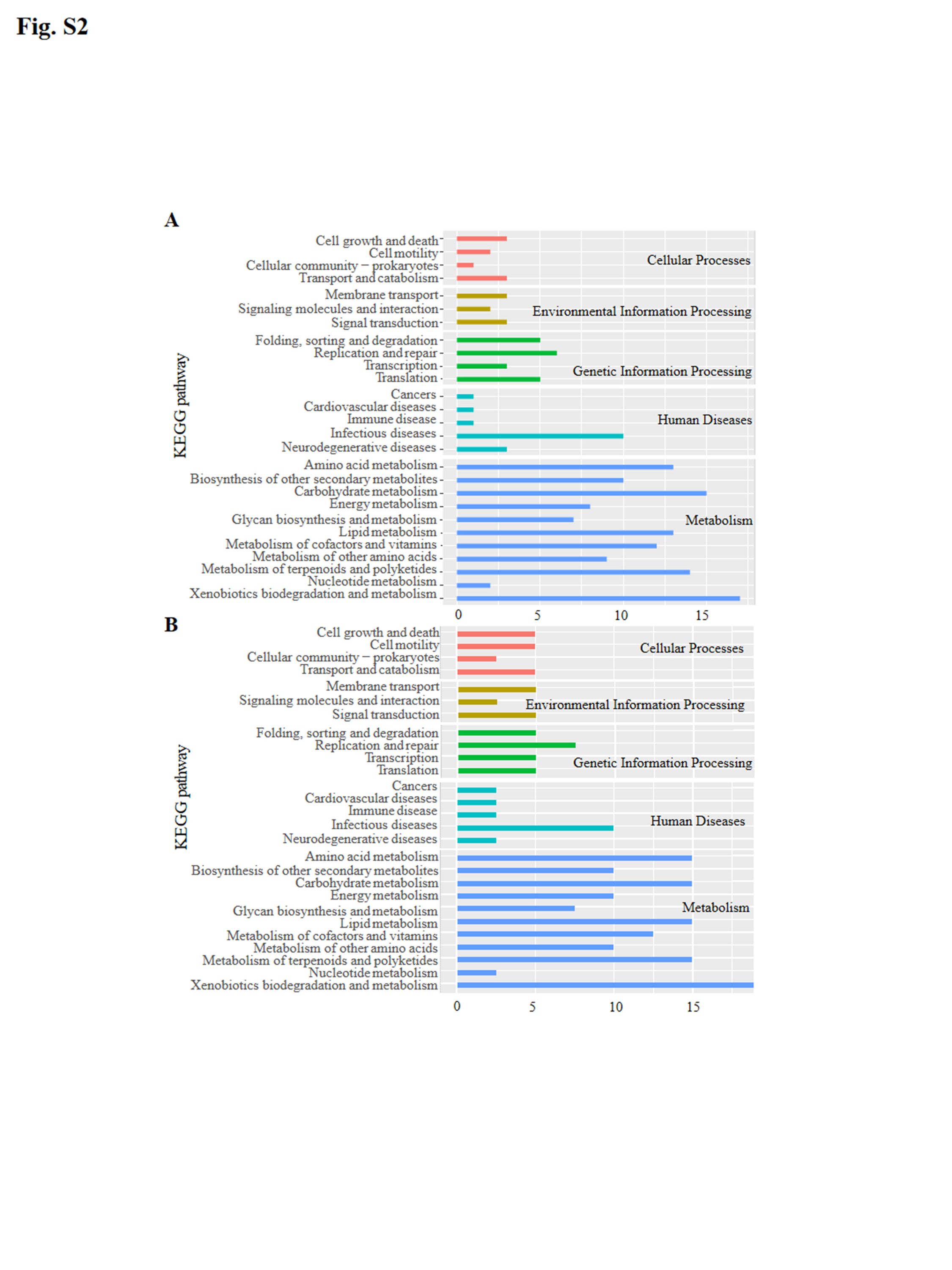

Supplement: Supplementary Figure 2 — Kyoto Encyclopedia of Genes and Genomes (KEGG) analysis of differentially expressed genes (DEGs) belonging to H_1 vs D_1 (A) and H_2 vs D_2 (B). Respective name of each KEGG pathway was shown on the left, and the pathway categories were indicated on the right. [file Image_2.TIF]

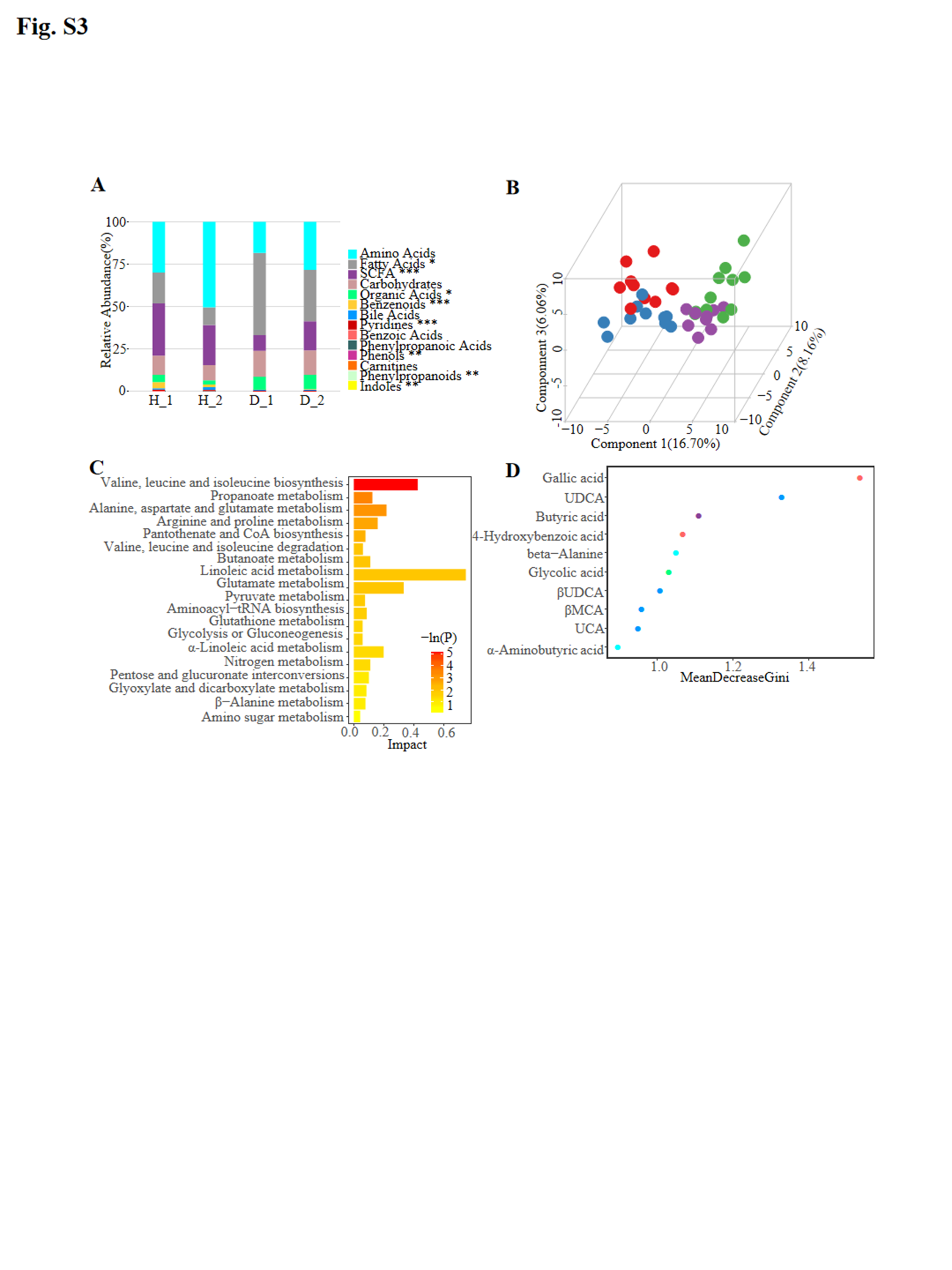

Supplement: Supplementary Figure 3 — Alterations of fecal metabolome profiles of neonatal calves post ESBL-EAEC infection. (A) The classifications of total metabolome compounds in H_1, H_2, D_1, and D_2 groups. The relative abundances of metabolites in different groups and the corresponding significant changes were shown. P values were determined using the nonparametric Kruskal-Wallis test. *p ≤ 0.05, **p ≤ 0.01, ***p ≤ 0.001. (B) Three-dimensional Partial Least Squares Discriminant Analysis (PLS-DA) was used here to cluster the fecal metabolome profiles of calves. The metabolome profiles for the H_1, H_2, D_1, or D_2 group was shown in the same color, respectively. (C) KEGG pathway enrichment analysis associated with the dramatically changed metabolites. Respective name of KEGG pathway was shown on the left, and the corresponding P value was shown on the right with a gradient color. P values were acquired following two-side Fisher's exact tests with Benjamini-Hochberg correction for multiple testing. (D) Differentiated metabolites were displayed using random forest supervised machine learning algorithm among H_1, H_2, D_1, and D_2 groups. Respective name of metabolite was shown on the left. Top 10 metabolites in fecal samples were shown with different colors, and the rank values are shown as Mean Decrease Gini. [file Image_3.TIF]

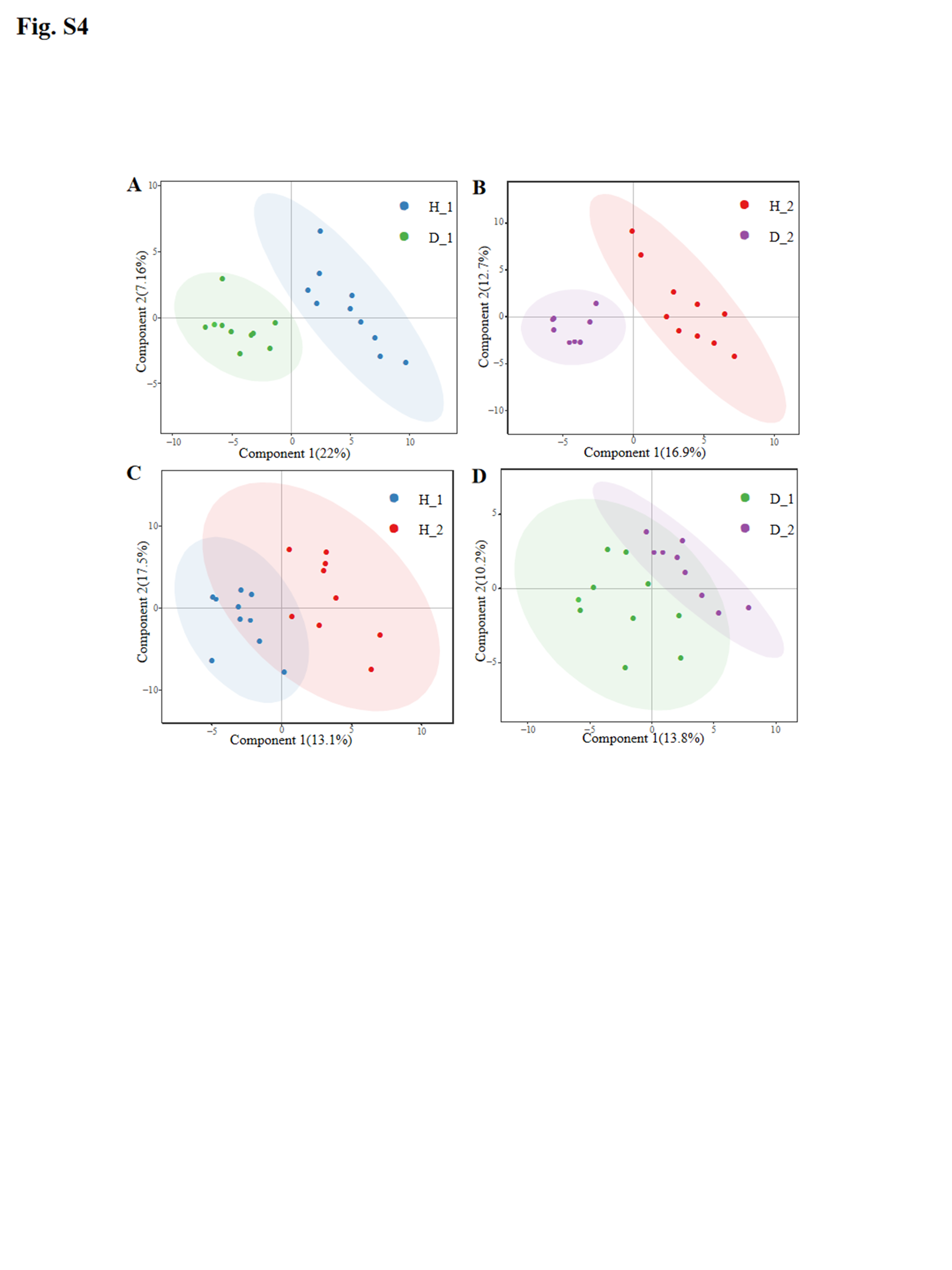

Supplement: Supplementary Figure 4 — Fecal samples cognate metabolomics analyses in healthy and diarrheic calves. PLS-DA for neonatal calves in H_1 vs D_1 (A), H_2 vs D_2 (B), H_1 vs H_2 (C), and D_1 vs D_2 (D). H, healthy calves; D, diarrheic calves. [file Image_4.TIF]

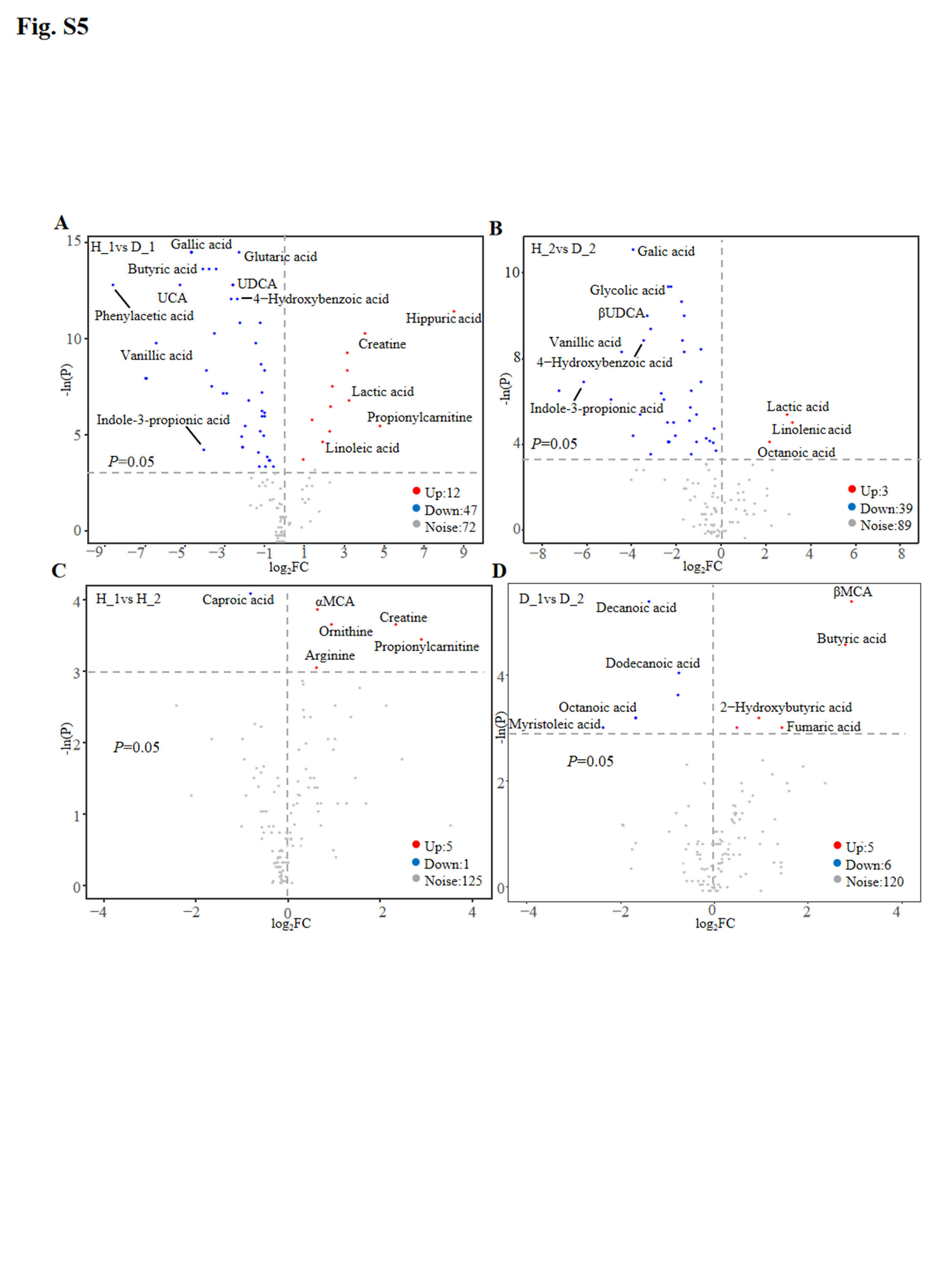

Supplement: Supplementary Figure 5 — Differentiated fecal metabolomics analyses from healthy and diarrheic calves representing by volcano maps. Enriched metabolites were identified by analyses with Fold Change (FC) values ≥1 (|log2FC|≥0) and P values of < 0.05. Significant upregulation and downregulation of metabolites were shown separately in red and blue. Metabolites with no obvious changes (Noise) were displayed in gray. The distinguished metabolites of H_1 vs D_1 (A), H_2 vs D_2 (B), H_1 vs H_2 (C), and D_1 vs D_2 (D) were respectively shown. Each counting numbers of upregulated or downregulated metabolites were shown on the right. [file Image_5.TIF]

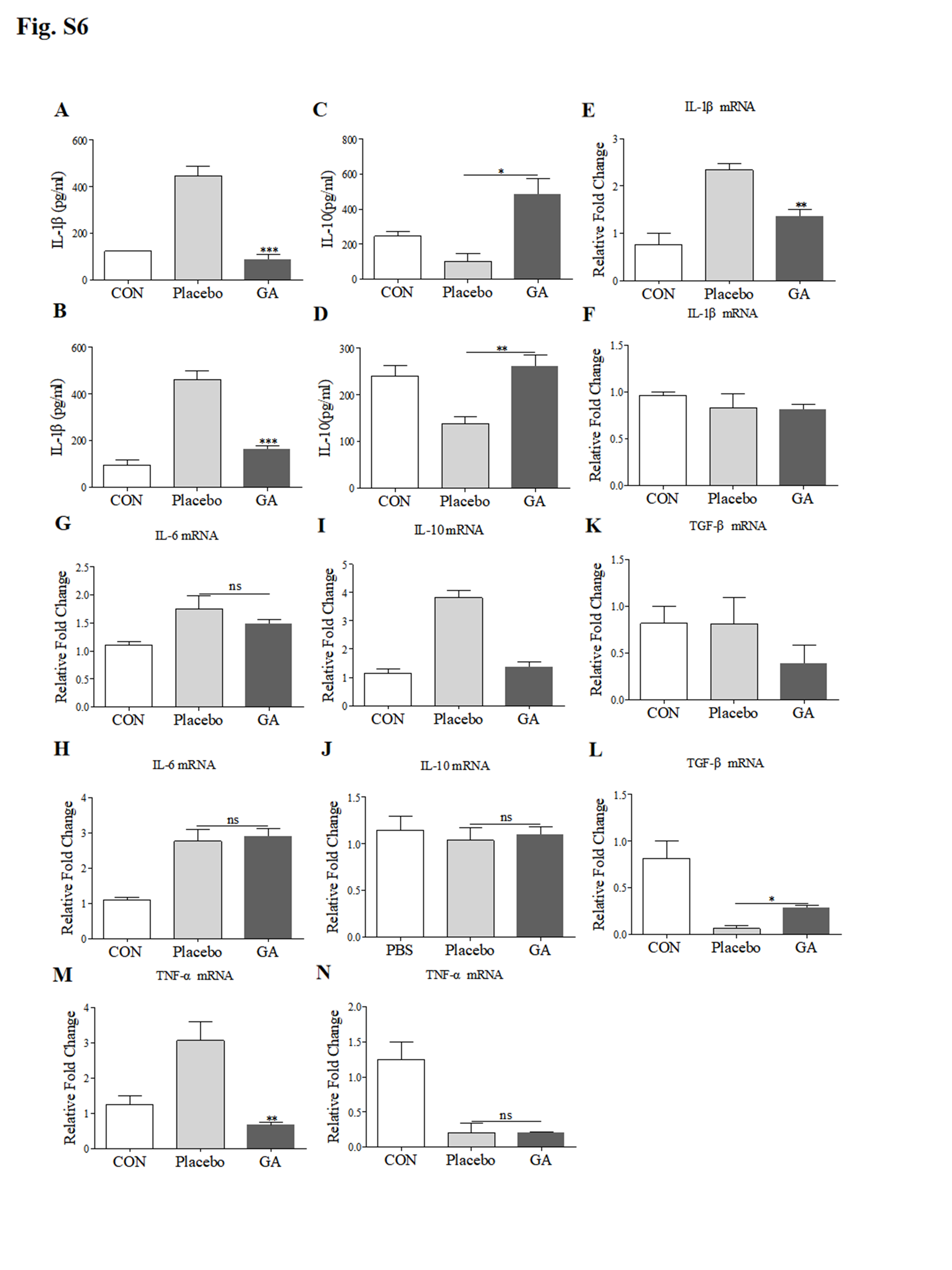

Supplement: Supplementary Figure 6 — Relative expression levels of inflammatory cytokines in neonatal mice peritonitis sepsis model. Serum levels of IL-1β (A,B) and IL-10 (C,D) assessed by ELISA. Colonic mRNA levels of five representative cytokines, namely, IL-1β (E,F), IL-6 (G,H), IL-10 (I,J), TGF-β (K,L), and TNF-α (M,N), assessed by qRT-PCR. Data were presented as means ± SEM. Statistical significance was analyzed using unpaired t-test. *p ≤ 0.05, **p ≤ 0.01, ***p ≤ 0.001. [file Image_6.TIF]

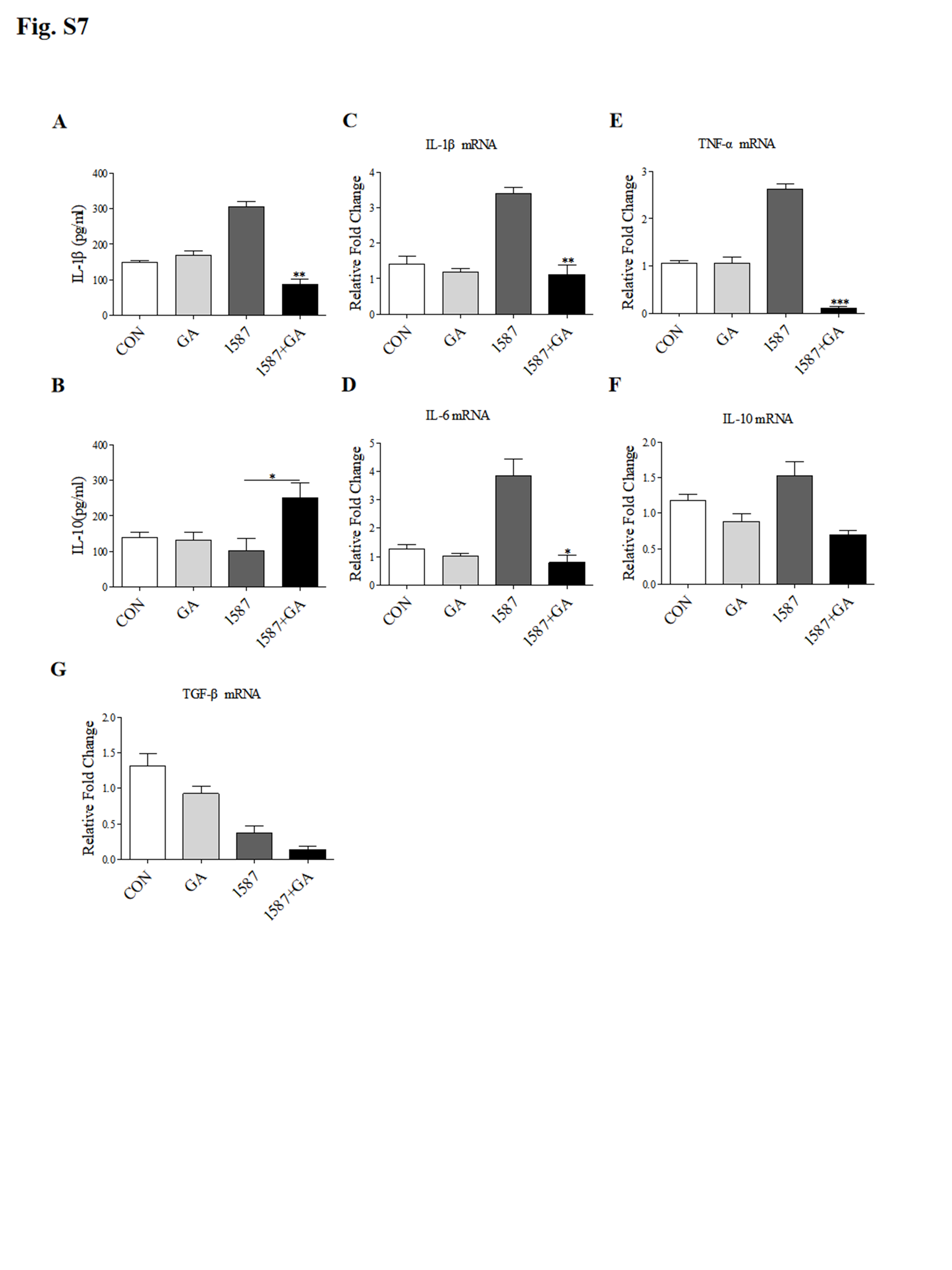

Supplement: Supplementary Figure 7 — Relative expression levels of inflammatory cytokines in neonatal mice oral infection model. Serum levels of IL-1β (A) and IL-10 (B) assessed by ELISA. Colonic mRNA expression levels of five representative cytokines, namely, IL-1β (C), IL-6 (D), TNF-α (E), IL-10 (F), and TGF-β (G), assessed by qRT-PCR. Data were presented as means ± SEM. Statistical significance was analyzed using unpaired t-test. *p ≤ 0.05, **p ≤ 0.01, ***p ≤ 0.001. [file Image_7.TIF]

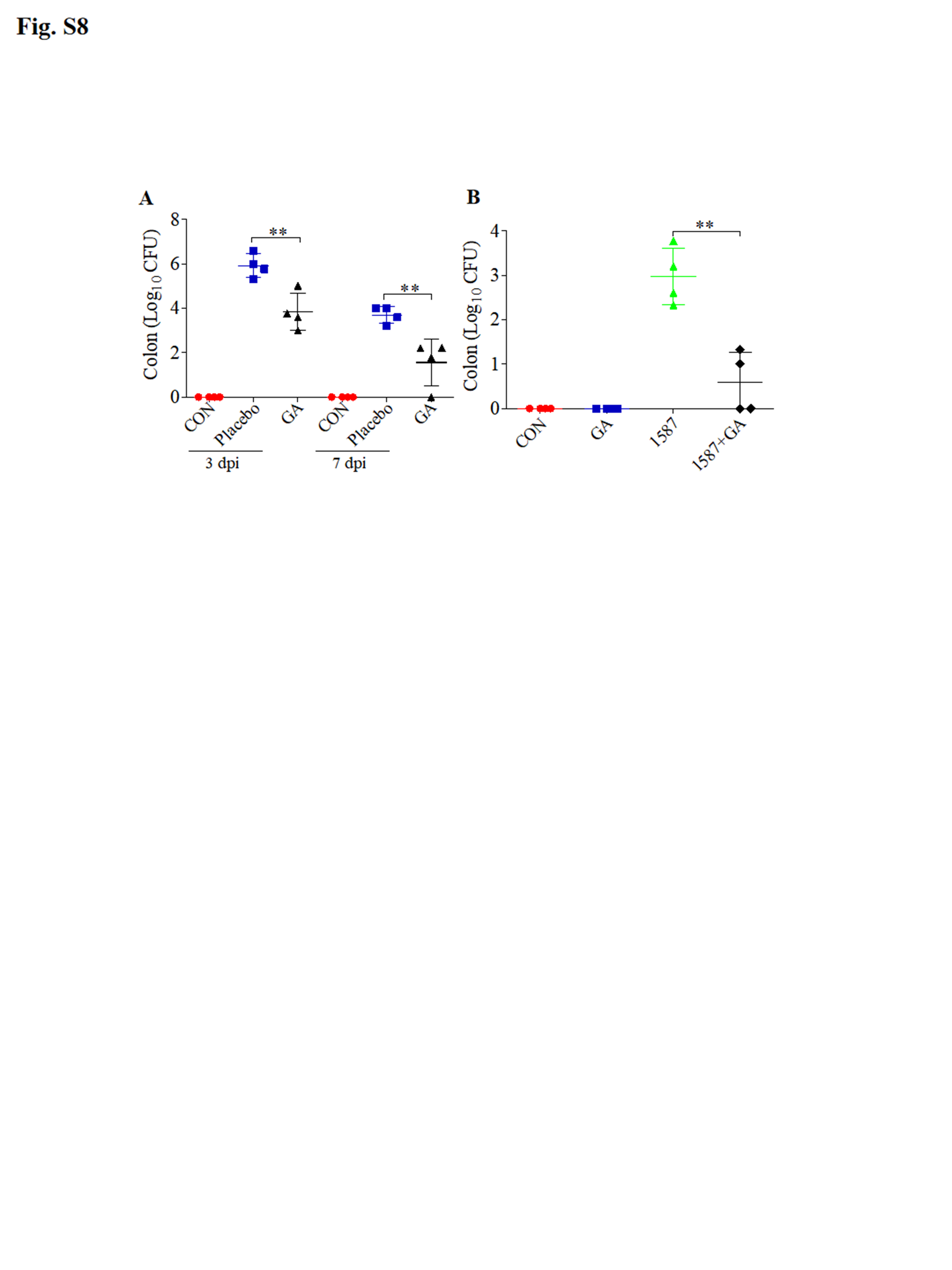

Supplement: Supplementary Figure 8 — The suppressive effect of oral gallic acid (GA) on E. coli 1587 colonization in neonatal mice sepsis (A) and oral infection (B) models. Data were presented as means ± SEM. Statistical significance was analyzed using unpaired t-test. *p ≤ 0.05, **p ≤ 0.01, ***p ≤ 0.001. [file Image_8.TIF]

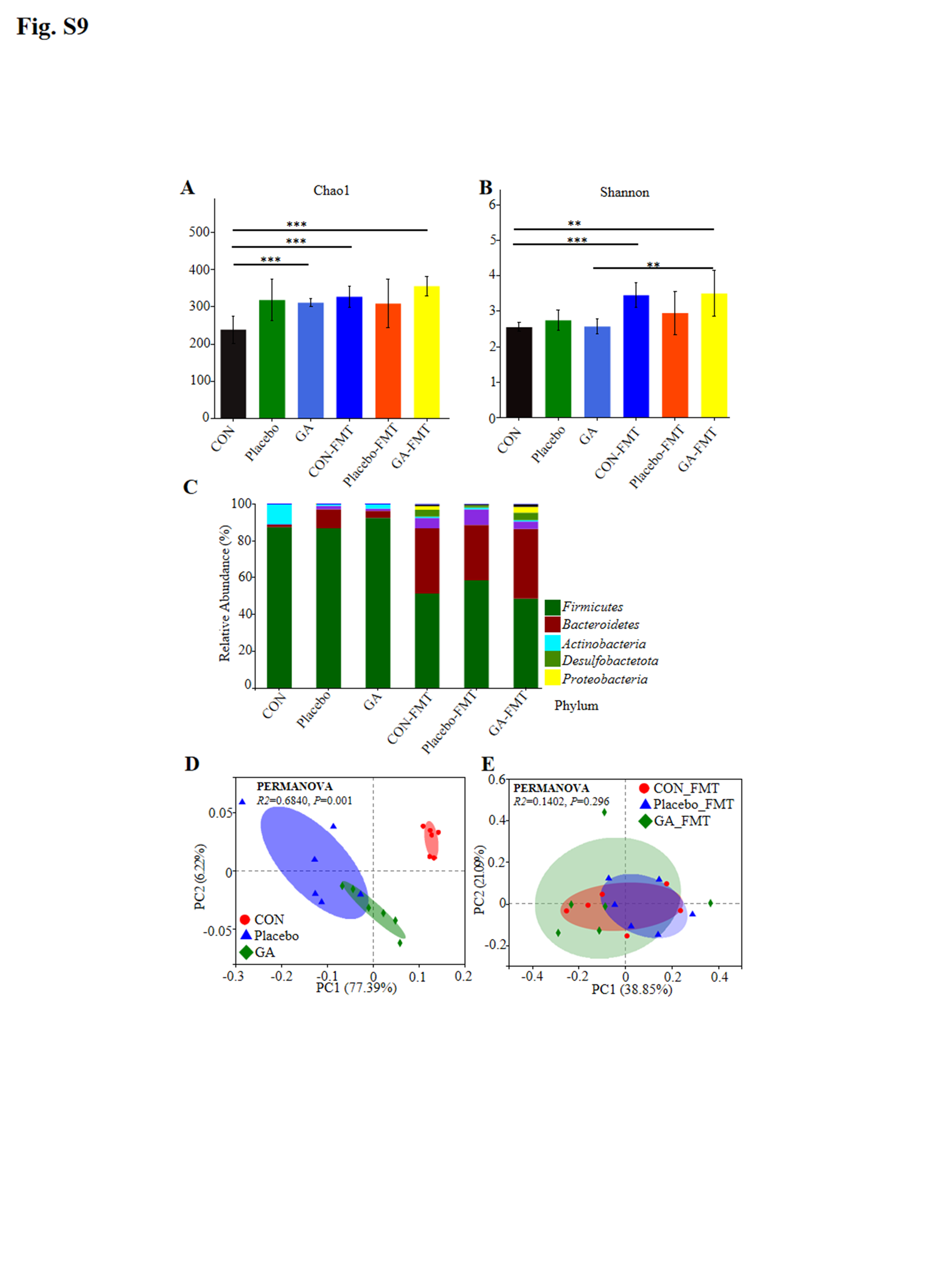

Supplement: Supplementary Figure 9 — Fecal microbiota transplantation (FMT) of oral GA mice reconstructed the hindgut microbiota in neonatal mice post infection. The α-diversity of different groups by chao1 (A) or shannon index (B). Data were shown as means ± SEM. Statistical significance was analyzed using unpaired t-test. *p ≤ 0.05, **p ≤ 0.01, ***p ≤ 0.001. (C) Mice fecal bacterial phylum's relative abundance represented by 99.5% of their community. Principal coordinate analysis (PCoA) of fecal bacteria based on the weighted UniFrac distance matrix of FMT donors (D) and recipients (E). The data were assessed using permutational ANOVA (PERMANOVA) analysis with 999 permutations. [file Image_9.TIF]

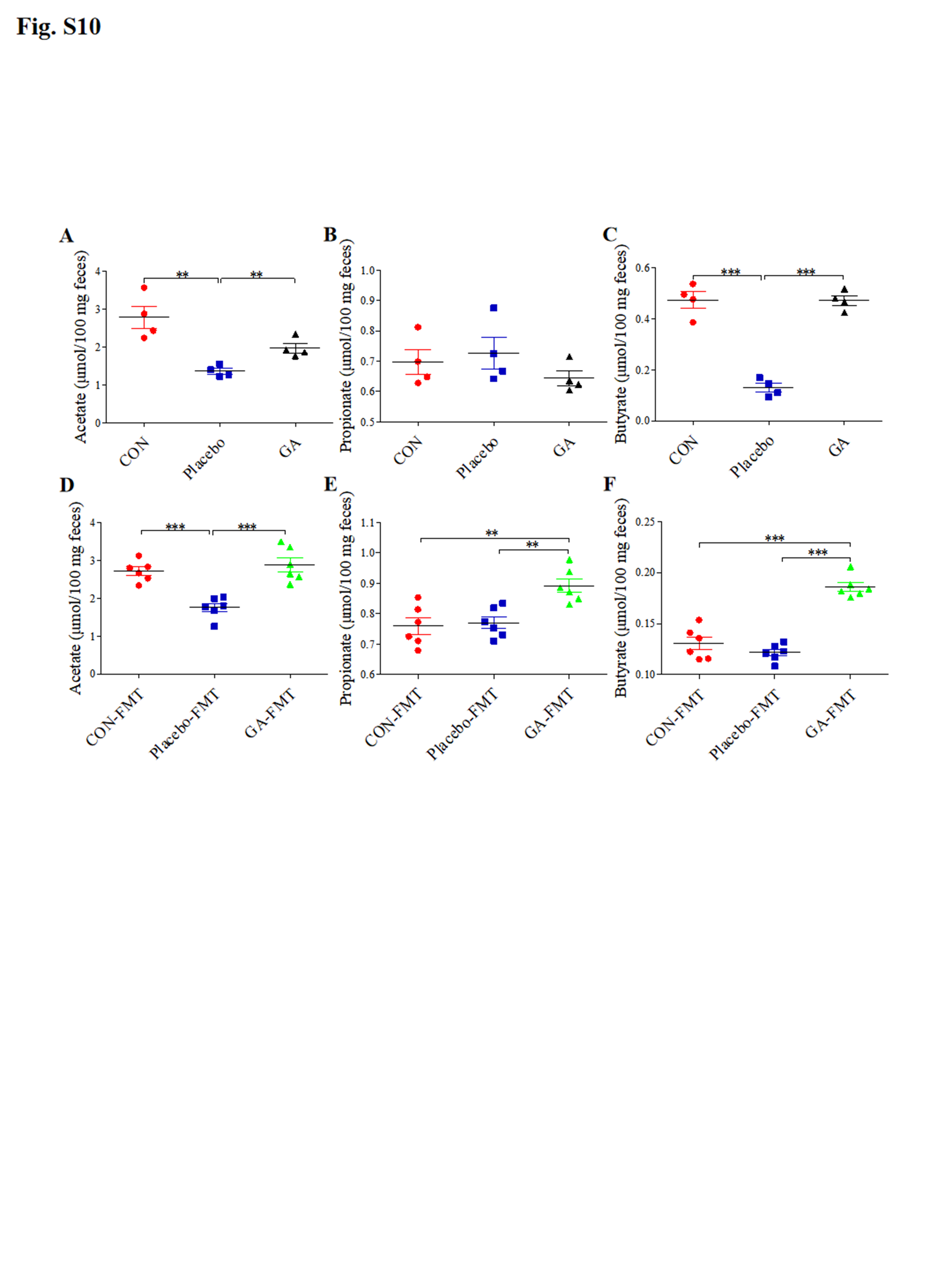

Supplement: Supplementary Figure 10 — Colonic short-chain fatty acid (SCFA) levels of neonatal mice donors and recipients. The concentrations of acetate (A), propionate (B), and butyrate (C) in donors upon oral GA therapy (n = 4 per group). Concentrations of acetate (D), propionate (E), and butyrate (F) in recipients upon FMT (n = 6 per group). Data were shown as means ± SEM. Statistical significance was analyzed using unpaired t-test. *p ≤ 0.05, **p ≤ 0.01, ***p ≤ 0.001. [file Image_10.TIF]

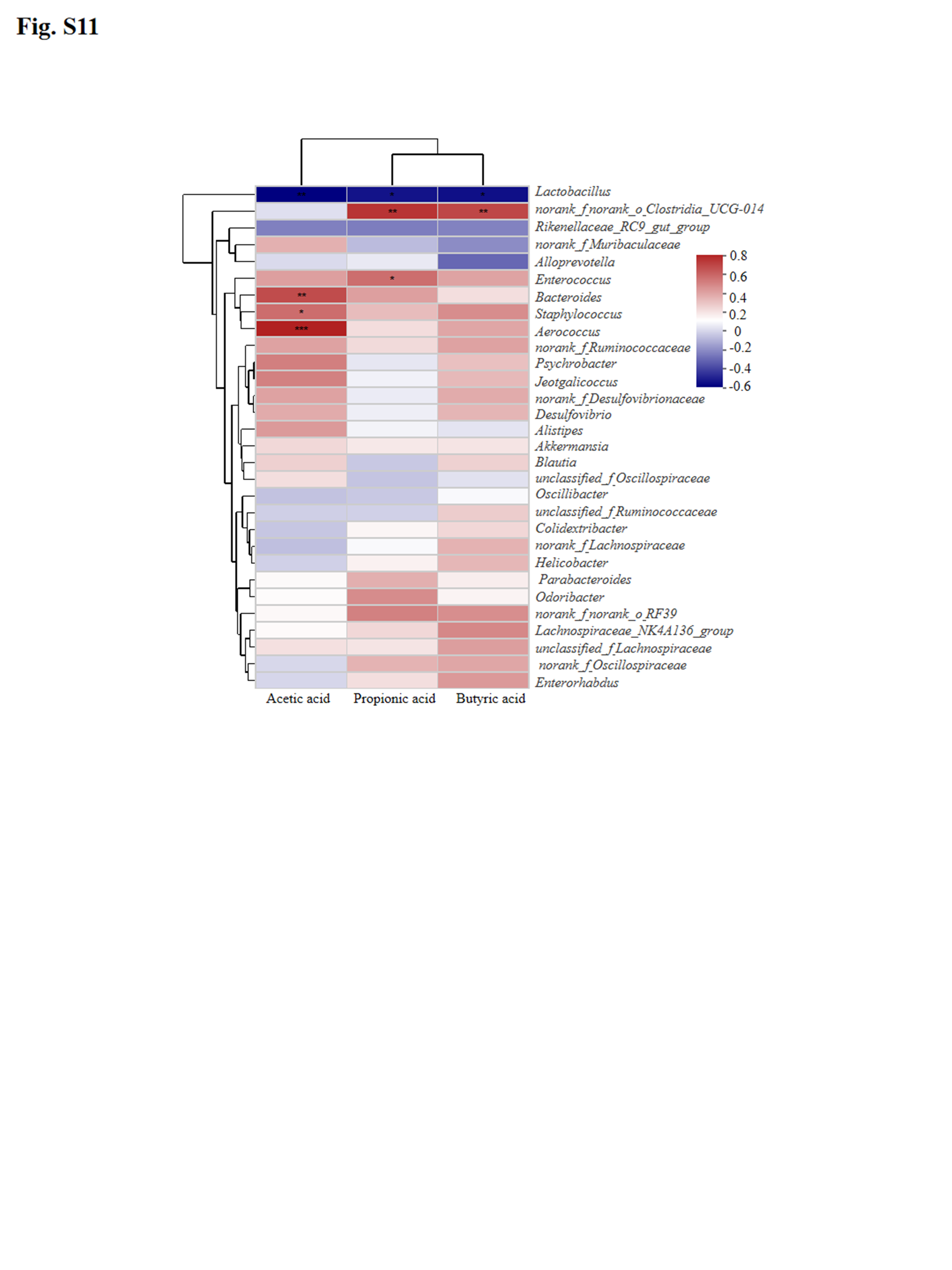

Supplement: Supplementary Figure 11 — Spearman correlation between fecal microbiota of FMT recipients and SCFA productions, including acetic acid, propionic acid, and butyric acid. The red color denoted a positive correlation, while blue color denoted a negative correlation. The intensity of the color was proportional to the strength of Spearman correlation. *p ≤ 0.05, **p ≤ 0.01, ***p ≤ 0.001. [file Image_11.TIF]
